# Peripheral inflammatory biomarkers are associated with cognitive function and dementia: Framingham Heart Study Offspring cohort

**DOI:** 10.1111/acel.13955

**Published:** 2023-08-16

**Authors:** Jiachen Chen, Margaret F. Doyle, Yuan Fang, Jesse Mez, Paul K. Crane, Phoebe Scollard, ADSP Data Harmonization Consortium Cognitive Harmonization Core, Claudia L. Satizabal, Michael L. Alosco, Wei Qiao Qiu, Joanne M. Murabito, Kathryn L. Lunetta

**Affiliations:** ^1^ Boston University School of Public Health Department of Biostatistics Boston Massachusetts USA; ^2^ Department of Pathology and Laboratory Medicine Larner College of Medicine, University of Vermont Burlington Vermont USA; ^3^ Boston University Chobanian & Avedisian School of Medicine, Boston University Alzheimer's Disease Research Center and CTE Center Boston Massachusetts USA; ^4^ Department of Neurology Boston University Chobanian & Avedisian School of Medicine Boston Massachusetts USA; ^5^ Framingham Heart Study, National Heart, Lung, and Blood Institute and Boston University Chobanian & Avedisian School of Medicine Framingham Massachusetts USA; ^6^ Division of General Internal Medicine, Department of Medicine University of Washington Seattle Washington USA; ^7^ University of Texas Health Science Center at San Antonio, Glenn Biggs Institute for Alzheimer's and Neurodegenerative Diseases San Antonio Texas USA; ^8^ Department of Psychiatry Boston University Chobanian & Avedisian School of Medicine Boston Massachusetts USA; ^9^ Boston University Chobanian & Avedisian School of Medicine Department of Pharmacology & Experimental Therapeutics Boston Massachusetts USA; ^10^ Department of Medicine, Section of General Internal Medicine Boston University Chobanian & Avedisian School of Medicine and Boston Medical Center Boston Massachusetts USA

**Keywords:** Alzheimer's disease, cognitive aging, peripheral inflammation, protein biomarkers

## Abstract

Inflammatory protein biomarkers induced by immune responses have been associated with cognitive decline and the pathogenesis of Alzheimer's disease (AD). Here, we investigate associations between a panel of inflammatory biomarkers and cognitive function and incident dementia outcomes in the well‐characterized Framingham Heart Study Offspring cohort. Participants aged ≥40 years and dementia‐free at Exam 7 who had a stored plasma sample were selected for profiling using the OLINK proteomics inflammation panel. Cross‐sectional associations of the biomarkers with cognitive domain scores (*N* = 708, 53% female, 22% apolipoprotein E (*APOE*) ε4 carriers, 15% *APOE* ε2 carriers, mean age 61) and incident all‐cause and AD dementia during up to 20 years of follow‐up were tested. *APOE* genotype‐stratified analyses were performed to explore effect modification. Higher levels of 12 and 3 proteins were associated with worse executive function and language domain factor scores, respectively. Several proteins were associated with more than one cognitive domain, including IL10, LIF‐R, TWEAK, CCL19, IL‐17C, MCP‐4, and TGF‐alpha. Stratified analyses suggested differential effects between *APOE* ε2 and ε4 carriers: most ε4 carrier associations were with executive function and memory domains, whereas most ε2 associations were with the visuospatial domain. Higher levels of TNFB and CDCP1 were associated with higher risks of incident all‐cause and AD dementia. Our study found that TWEAK concentration was associated both with cognitive function and risks for AD dementia. The association of these inflammatory biomarkers with cognitive function and incident dementia may contribute to the discovery of therapeutic interventions for the prevention and treatment of cognitive decline.

## Graphical abstract

In this study, we examined the association between OLINK inflammatory protein biomarkers and cognitive function and incident dementia outcomes in the Framingham Heart Study Offspring cohort. Apolipoprotein E genotype‐stratified analyses were also conducted to identify associations in specific strata. We found several proteins associated with cognitive function and risks for incident dementia: IL10, LIF‐R, TWEAK, CCL19, IL‐17C, MCP‐4, TGF‐alpha, TNFB, CDCP1, etc.

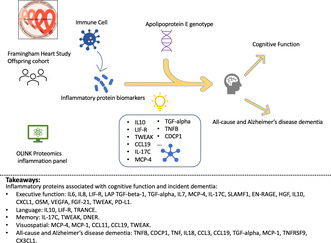

**Abbreviations**
AD: Alzheimer's disease
ADRD: Alzheimer's disease and related dementias
APOE: apolipoprotein E
AF: atrial fibrillation
CVD: cardiovascular disease
CI: confidence interval
EF: executive function
FDR: false discovery rate
FHS: Framingham Heart Study
HR: hazard ratio
HVOT: Hooper Visual Organization Test
LAN: language
LOD: limit of detection
MEM: memory
MMSE: Mini‐Mental State Examination
NP: neuropsychological
SD: standard deviation
VIS: visuospatial

## 1 INTRODUCTION

Alzheimer's disease (AD) and AD‐related dementias (ADRD) are projected to double by 2060 in the United States so an estimated 13.9 million adults aged 65 years and older will suffer from the disease (Matthews et al., 2018). ADRDs are multifactorial in etiology, with clinical stages occurring over long periods of time. Neuroinflammation is increasingly recognized as a contributor to ADRD pathogenesis and can be induced by both central nervous system intrinsic factors and systemic factors outside the brain (Heppner et al., 2015; Schwartz & Deczkowska, 2016). Further, genetic association studies of AD have identified several genes encoding proteins implicated in innate and adaptive immunity (Jorfi et al., 2023). However, it is largely unclear which circulating inflammatory factors may provide opportunities for the development of new diagnostic tests and become potential drug targets.

Meta‐analyses of relatively large samples across multiple countries have demonstrated associations between a limited number of peripheral inflammatory biomarkers including CRP and IL‐6 among others and all‐cause dementia, but not AD dementia (Darweesh et al., 2018; Koyama et al., 2013). However, there are conflicting results as another meta‐analysis reported associations of several inflammatory markers with AD dementia including CRP, IL‐6, IL‐1β, TNFR1, TNFR2, α1‐ACT, CD40L, IL‐8, and MCP‐1 (Shen et al., 2019). Among individuals with AD dementia, peripheral inflammatory biomarkers (IL‐8, MIP‐1B, MPO, NGAL, TNF) also predicted a decline in executive function (Bawa et al., 2020). In the Framingham Heart Study (FHS), chronic peripheral inflammation measured with CRP increased the risk for dementia and AD dementia but only in apolipoprotein E (*APOE*) ε4 carriers (Tao et al., 2018). Data from the Alzheimer's Disease Neuroimaging Initiative (ADNI) cohort also showed that plasma biomarker profiles differed across *APOE* genotype carrier groups in patients with AD dementia, mild cognitive impairment, and cognitively normal participants (Soares et al., 2012). CRP and gamma interferon plasma protein were lowest and IL‐13 levels were highest among ε4 carriers compared with other genotype groups. Further investigation in larger cohorts is needed to confirm results by *APOE* carrier status and to account for important potential confounders.

A limited number of peripheral inflammatory biomarkers have also been linked to cognitive function outcomes. In the Northern Manhattan Study, higher levels of IL‐6 were associated with a global measure of cognition (Mini‐Mental State Examination total scores) and with cognitive decline (Economos et al., 2013), while no association was observed for IL‐1, IL‐2, TNF‐α, and the corresponding receptors IL‐2R, TNFR‐1, and TNFR‐2 (Wright et al., 2006). The MEMO study, a study of community‐dwelling older adults, identified an association between higher levels of IL‐8 and worse memory and processing speed, but no association with other cytokines (IL‐1beta, sIL‐4R, IL‐6, IL‐10, IL‐12, and TNF‐α) (Baune et al., 2008). Higher levels of IL‐6 have been associated with poorer performance on cognitive testing and with cognitive decline in diverse groups of older men and women (Palta et al., 2015; Singh‐Manoux et al., 2014; Yaffe et al., 2003). A recent report identified CCL11 (eotaxin), CXCL9, hepatocyte growth factor (HGF), and serpine 1 associations with multiple cognitive domains and overall cognition that remain to be validated in other studies (Elkind et al., 2021). Despite several reports linking proinflammatory factors to cognitive decline, few have identified circulating inflammatory markers that have a protective effect on cognitive functions. Due to the limited overlap of measured circulating biomarkers across studies, different study designs, and conflicting association results, further exploration is needed in population‐based samples and a broad selection of inflammatory proteins to investigate the association between inflammation and cognitive function, while accounting for *APOE* genotype status.

The aim of this study was to investigate the association of circulating inflammatory biomarkers measured from the OLINK inflammation panel with cognitive function as well as incident all‐cause and AD dementia in the community‐based FHS Offspring cohort. We hypothesized that several inflammatory biomarkers would be associated with measures of cognitive function cross‐sectionally, as well as incident all‐cause and AD dementia. Given that risks for dementia and cognitive decline and peripheral biomarker signatures may both be influenced by *APOE* genotype carrier status, we also examined the association between inflammatory biomarkers and cognitive outcomes in different *APOE* strata. We hypothesized that we would identify unique biomarker‐cognitive outcome associations for different *APOE* genotypes.

## 2 METHODS

### 2.1 Study sample

The FHS Offspring cohort began in 1971 when 5214 offspring of the FHS Original cohort and spouses of the Offspring were recruited (Feinleib et al., 1975). The FHS Offspring participants have been examined every 4–8 years since enrollment, have undergone neuropsychological (NP) testing every 5–6 years since 1999, and are under continuous surveillance for the onset of dementia (Satizabal et al., 2016). Details regarding sample inclusion and exclusion for the cross‐sectional NP testing outcome sample and incident dementia outcomes sample are provided in Figure S1. We identified a sample of 879 Offspring participants who were at least 40 years old and dementia‐free at FHS Offspring Exam 7 (1998–2001) with an existing stored plasma sample for inflammatory biomarker profiling and 877 of these samples with complete biomarker levels passed the quality control processes. We excluded eight participants without *APOE* genotyping leaving 869 participants with complete *APOE* genotype and inflammatory biomarker protein levels available for our analyses. An additional 161 participants missing neuropsychological testing within 5 years after their Exam 7 visits were excluded from the NP test association analyses, leaving a final sample of 708 participants. For the incident all‐cause and AD dementia analyses, 32 participants with prevalent stroke, missing outcomes, or covariate data were excluded, leaving a sample of 837 participants. Written informed consent from participants was obtained at every attended examination. The study protocol and examinations were reviewed and approved by the Institutional Review Board at Boston University Medical Center.

### 2.2 OLINK inflammation panel

The OLINK Inflammation panel measured 92 protein biomarkers using existing fasting plasma samples collected at the Offspring Exam 7 that had been stored at ‐80C. The OLINK reagents were based on the Proximity Extension Assay (PEA) technology where 92 oligonucleotide‐labeled antibody probe pairs bind to their respective target proteins. The protein expression levels were represented by Normalized Protein eXpression (NPX) units, measuring a relative quantification unit on $\log_{2}$ scale, with one NPX difference indicating a doubling of protein concentration (Kuan et al., 2020). The OLINK NPX Signature software was used for quality control and normalization of data. More information is available online (https://www.OLINK.com). Phantoms (duplicate participant samples) and OLINK plate controls were included as part of quality control and calculations of the limit of detection (LOD) for proteins (Kuan et al., 2020).Samples were randomly assigned to 11 plates and were run in a single batch at OLINK. The coefficient of variation across plates within all protein were below 5% so that there were no plate effects. The full list of protein biomarkers included in the OLINK inflammation panel and the corresponding percentage of samples missing or with values below LOD are provided in Table S1. Proteins with >50% of participant values below LOD were excluded from analyses, a value suggested by OLINK and adopted by others (Costi et al., 2021; Drake et al., 2021; Harlid et al., 2021) leaving a total of 68 proteins for downstream analyses (see Table S2a). According to the OLINK guidelines, we used the actual data value below LOD for the subset of proteins with values below LOD (Maglinger et al., 2021), as LOD is a conservative measurement in large multi‐plate studies and using the observed values may improve statistical power. We performed rank‐based inverse normal transformations on all proteins to standardize and reduce skewness (Folkersen et al., 2020).

### 2.3 Neuropsychological tests and factor scores

FHS Offspring participants began NP testing in 1999. Participants were administered an NP test battery according to standard test administration protocols by trained examiners, as part of a study investigating brain structure and cognition in the FHS Offspring Cohort (Au et al., 2004). We identified a total of 708 participants who were at least 40 years old and dementia‐free at Exam 7, had *APOE* genotyping, and underwent NP tests within 5 years after providing a plasma sample at Exam 7 (Figure S1).

Factor scores for three cognitive domains—memory (MEM), executive function (EF), and language (LAN), were developed based on NP test battery using data across all FHS NP testing visits (Scollard et al., 2023). Items from Mini‐Mental State Examination (MMSE) or Consortium to Establish a Registry for Alzheimer's Disease (CERAD) also contributed to these domain scores. Each domain was calibrated using bi‐factor confirmatory factor analysis models with scores obtained for each participant at each visit. With this approach, the individual NP tests can be summarized into a domain factor score, which makes comparisons straightforward even if the cognitive tests taken by each participant in FHS are different (e.g., if a participant did not take all of the tests in one of the domains), minimizing ceiling effects, and facilitates comparisons across cohorts (Scollard et al., 2023). There were not enough visuospatial (VIS) domain items to enable full calibration of scores for that domain, therefore, we used rank‐normalized Hooper Visual Organization Test (HVOT) test scores to represent that domain.

Apart from the four domain scores, we include association results for a subset of six individual NP tests in the supplement to this study: the Logical Memory‐Delayed Recall, Paired Association Learning, and Visual Reproduction‐Delayed Recall (LMD, PASD, and VRD); the difference between Trail Making Tests Parts B and A (TRAILSBA) and the Similarities subtest (SIM); and 30‐item version of the Boston Naming Test (BNT30).

Some participants participated in NP tests at multiple time points. Our analyses used the cognitive scores obtained from the first NP testing visit after the participant's Exam 7 core visit, the exam at which the blood sample for the biomarker panel was drawn. We excluded individuals who did not have an NP testing visit within 5 years after Exam 7.

### 2.4 FHS dementia ascertainment

The cognitive status of participants was monitored by administering subjective memory questions, serial MMSE testing, and NP tests every 5–6 years, adjusted for education level and previous performance (Satizabal et al., 2016). Participants who performed poorly were suspected to have cognitive impairment and were invited for further assessment. For example, participants with a decline of more than three points between consecutive MMSE tests, a decrease of more than five points compared with any previous test, or MMSE less than or equal to 24 were identified as people who might have dementia and they were therefore invited to undergo additional examinations (Seshadri et al., 1997). Assessments would also be performed on participants who self‐reported or whose family members reported cognitive decline, participants who were referred by physicians or FHS investigators, and participants who were identified after reviewing outside medical records (Satizabal et al., 2016). Evaluations were conducted by a neurologist and a neuropsychologist and participants raising concerns for dementia were sent to a review committee to make consensus decisions regarding the presence of dementia, dementia type, and year of onset (Seshadri et al., 1997). Criteria for the diagnosis of dementia were based on the Diagnostic and Statistical Manual of Mental Disorders, fourth edition (DSM‐IV) (Satizabal et al., 2016). The diagnosis of AD dementia was established based on the National Institute of Neurological and Communicative Disorders and Stroke (NINCDS) and the Alzheimer's Disease and Related Disorders Association (ADRDA) (McKhann et al., 1984; Seshadri et al., 1997).

### 2.5 Covariates for association analyses

Participants' age and sex were ascertained during the same exam visit (Offspring Exam 7) as the blood plasma sample. *APOE* genotypes were determined using polymerase chain reaction and restriction isotyping (Lahoz et al., 2001). The education level recorded at the time of the NP test was used for analyses with cognitive scores. The highest education level recorded during the participants' lifetime was used for the analyses of dementia outcomes.

### 2.6 Statistical analyses

In all analyses, the protein levels and the NP tests were inverse‐normal transformed to reduce skewness and have a distribution with a mean of 0 and standard deviation (SD) of 1. For each outcome, we conducted a combined analysis for the full sample adjusting for *APOE* genotype and stratified analyses within three subgroups defined by *APOE* genotype status. In the combined analysis, we used additive coding for the number of *APOE* ε2 and ε4 alleles. For the stratified analyses, we excluded individuals with *APOE* ε2ε4 genotype and defined participants according to *APOE* carrier status into three subgroups: *APOE* ε2 carrier (ε2ε2 and ε3ε2 genotypes), *APOE* ε4 carrier (ε4ε4 and ε3ε4 genotypes), and *APOE* ε3ε3 genotype (the reference group). In all analyses, the education level was a four‐category variable with levels: did not graduate high school, high school graduate, attended some college, or college graduate. All regression models accounted for familial relationships via the kinship coefficient matrix.

#### 2.6.1 Association of NP cognitive scores with inflammatory proteins

Our primary analyses focused on the three cognitive domain factor scores and HVOT for the VIS domain. Analyses for six individual NP tests are included in the Supplementary Files. We tested each of the 68 proteins (predictor) for association with each of the domain scores (outcome) individually using linear mixed‐effects regression. The primary model (Model 1) included sex, age, education level, time in years between Exam 7 (blood sample) and cognitive testing date, a retest indicator suggesting whether the cognitive function test was the first one taken by this participant to account for practice effects, and *APOE* genotype. We used a secondary model (Model 2) to assess the robustness of results after accounting for prevalent cardiovascular disease (CVD) and CVD risk factors. Model 2 incorporated all covariates in Model 1 as well as (1) indicators for prevalent stroke, prevalent CVD, and prevalent atrial fibrillation (AF) at Exam 7 and (2) CVD risk factors including systolic and diastolic blood pressures (mmHg), diabetes status, treatment for hypertension, body mass index (kg/m^2^), current smoking status, total cholesterol level (mg/dL), high‐density lipoprotein cholesterol levels (HDL, measured in mg/dL), and use of lipid‐lowering agents at Exam 7 (Fang, Doyle, Alosco, et al., 2022; Fang, Doyle, Chen, et al., 2022). Diabetes status was defined by whether any of the following was satisfied: use of antidiabetic medications, fasting blood glucose level ≥126 mg/dL, or random blood glucose level ≥198 mg/dL. Prevalent CVD was determined based on previous diagnosis before Exam 7 of coronary heart disease (myocardial infarction, angina pectoris, coronary insufficiency), transient ischemic attack, intermittent claudication, and congestive heart failure adjudicated by a panel of senior investigators (Lloyd‐Jones et al., 2006). We conducted the stratified analyses within each *APOE* status stratum with the same Model 1 and Model 2 covariates.

#### 2.6.2 Association of dementia outcomes with the inflammatory proteins

After excluding participants with prevalent stroke, we used Cox proportional hazard models to test for associations between each of the 68 proteins and the incidence of all‐cause and AD dementia separately, using the same covariates in Model 1 and Model 2 as used for the cognitive score outcome models except for the stroke indicator. Similar to the analysis with cognitive scores, we conducted *APOE*‐stratified analyses.

Full follow‐up from the date of Exam 7 through the year 2021 was recorded for participants, with a maximum follow‐up time of around 20.6 years and a median follow‐up time of 18.2 years. For participants with incident all‐cause dementia, the follow‐up time was defined by years from the baseline Exam 7 to the diagnosis of dementia. Participants who did not develop dementia were censored at the date of death, at the date of the last contact, or at the last date that they were known not to have dementia if lost to follow‐up. For participants with incident AD dementia, the follow‐up time was defined by years from the baseline Exam 7 to the diagnosis of AD dementia. Participants who did not develop AD dementia were censored at the onset date of other types of dementia, at the date of death, or at the date of the last contact through the year 2021.

For the cognitive function and dementia outcomes, we report effect sizes and hazard ratios, respectively, and their respective 95% confidence intervals (CIs) for each protein. As outcomes and predictors are standardized, effects are reported in SD units.

The false discovery rate (FDR) (Benjamini & Hochberg, 1995) was used to control the false rejections of true null hypotheses for each outcome and stratum separately, and FDR ≤ 0.1 was set as the threshold to declare significant associations within each set of analyses (68 pairwise associations with one outcome). All the statistical analyses were implemented in *R‐4.2.1* software (R Core Team, 2013). The linear mixed‐effect models were conducted using the *lmekin* function in the *coxme* package (Therneau & Therneau, 2015). The Cox proportional hazards models were conducted using the *coxph* function in *survival* package (Therneau & Lumley, 2015).

### 2.7 Sensitivity analyses

To further assess the robustness of the association analyses, we conducted a series of sensitivity analyses. First, the associations between proteins and cognitive function outcomes were evaluated through Model 1 including only the participants who underwent NP tests within two years after Exam 7, rather than 5 years. Second, to assess whether a subgroup of participants drove the NP associations, we investigated analyses through Model 1 excluding 14 participants with prevalent stroke, and additionally excluding individuals with prevalent chronic leukemia or lymphoma, who reported use of glucocorticoids at Exam 7, and who were identified as outliers by principal component analysis (PCA) based on the 68 rank‐normalized proteins. Third, as dementia is rare among younger participants, we assessed the associations between proteins with incident all‐cause and AD dementia on the subsamples with age at Exam 7 older than 60 years old using Model 1. We expected similar robustness for associations through Model 2, and thus did not present sensitivity analysis using Model 2 for this study.

## 3 RESULTS

### 3.1 Participant characteristics

Table 1 presents the demographic characteristics of the participants in the two analysis samples: the sample for cross‐sectional analyses with cognitive scores, and the sample used to investigate incident dementia. The two samples have an overlap of 688 individuals and share similar demographic characteristics: the average age was 61 years old at Exam 7, around 52% of the participants were female, the median MMSE score was 29, and the proportion of *APOE* ε2 and ε4 carriers was around 15% and 22%, respectively. In the dementia sample, 76% of the participants attended college, while the proportion was slightly higher (78%) in the NP sample.

**TABLE 1:** Participant characteristics at Exam 7, time of plasma sample for inflammatory biomarker measurement.

	NP/factor score sample	Dementia sample
Sample size, *n*, *n* (%)	708	837
Female, *n* (%)	372 (52.5%)	436 (52.1%)
Age, mean (range)	61 (40, 88)	61 (40, 88)
*APOE* ε2 carriers, *n* (%)	107 (15.1%)	122 (14.6%)
*APOE* ε4 carriers, *n* (%)	153 (21.6%)	184 (22.0%)
Attended college, *n* (%)	558 (78.8%)	637 (76.1%)
Current smoker, *n* (%)	85 (12.0%)	107 (12.8%)
BMI Kg/m^2^, mean (sd)	28 (5)	28 (5)
SBP mmHg, mean (sd)	126 (18)	126 (18)
DBP mmHg, mean (sd)	74 (10)	74 (9)
Hypertension Rx, *n* (%)	226 (31.9%)	281 (33.6%)
Total cholesterol mg/dL, mean (sd)	199 (37)	199 (37)
Triglycerides mg/dL, mean (sd)	135 (81)	137 (84)
Lipid Rx, *n* (%)	150 (21.2%)	190 (22.7%)
Fasting blood glucose mg/dL, mean (sd)	104 (27)	104 (27)
Type 2 diabetes Rx, *n* (%)	37 (5.2%)	49 (5.9%)
MMSE, median (IQR)	29 (2)	29 (2)
≥1 parent with dementia Dx, *n* (%)	153 (21.6%)	179 (21.4%)
Incident dementia through 2021, *n* (%)	65 (9.2%)	87 (10.4%)
Blood cancer prevalent Exam 7, *n* (%)	6 (0.8%)	6 (0.7%)
Blood cancer through 2019, *n* (%)	29 (4.1%)	32 (3.8%)
NP data within 5 years after Exam 7, *n* (%)	708 (100%)	688 (82.2%)
Time difference between NP test and Exam 7, mean (sd)	0.80 (0.79)	__
First NP test, *n* (%)	694 (98.0%)	__
Date of Exam 7 blood draw (yyyy/mm/dd)	1998/09/14–2001/10/26	1998/09/14–2001/10/26

Abbreviations: *APOE*, apolipoprotein E; BMI, body mass index; DBP, diastolic blood pressure; IQR, interquartile range; MMSE, Mini‐Mental State Examination; NP, Neuropsychological; SBP, systolic blood pressure; sd, standard deviation.

The mean and SD of the 68 proteins did not differ between the NP test participants and the dementia participants (Table S2a). Table S2b shows the mean (SD) of cognitive domain and NP scores and age for the cross‐sectional analysis and the number of events for the incident outcomes.

### 3.2 Overall summary

A summary of the numbers and direction of association of significantly associated proteins for the primary analyses of NP sample and dementia sample are illustrated in Tables 2 and 3. Proteins for which higher levels were associated with higher (better) cognitive scores are marked with a + sign to indicate a positive association while proteins for which higher levels are associated with lower (worse) cognitive scores are marked with a − sign to indicate a negative association. Most of the significant associations are negative, and more associations were observed for the executive function domain compared with the memory and language domains (Table 2). All the significant associations with incident all‐cause and AD dementia in the full sample were negative, meaning that higher protein levels were associated with a higher risk of the outcome (Table 3). The summary of significant proteins for the secondary analyses adjusting for Model 2 covariates are presented in Tables S3 and S4. While similar conclusions were observed, fewer significant associations remained after adjusting for cardiovascular risk factors for the full sample and the ε4 and ε3 strata. For the ε2 stratum, the number of proteins associated with cognitive domain scores was 6 from Model 1 but 14 for Model 2 which also adjusted for CVD and risk factor covariates.

**TABLE 2:** Summary of significant cross‐sectional associations between inflammatory biomarker proteins and cognitive domain scores using Model 1 in full sample and by *APOE* status strata.

Domain	Full sample	ε2 stratum	ε4 stratum	ε3 stratum
Executive Function	−:12	+:1	+:1 −:4	−:6
Language	−:3			
Memory			+:2 −:1	
Visuospatial		−:5		

*Note*: Model 1 covariates included: sex, age, education level, time in years between Exam 7 (blood sample) and cognitive testing date, a retest indicator, and *APOE* genotype.

Abbreviation: *APOE*, apolipoprotein E.

**TABLE 3:** Summary of significant associations for dementia outcomes using Model 1 in full sample and by *APOE* status strata.

Outcome	Full sample	ε4 stratum	ε3 stratum
Incident all‐cause dementia (Full)	−:2	−:8	−:3
Incident AD dementia (Full)	−:1	−:1	
Incident all‐cause dementia (>60 years old)	−:2 +:1	−:1	−:2
Incident AD dementia (>60 years old)	−:1 +:1	−:1	

*Note*: Model 1 covariates included: sex, age, education level, and *APOE* genotype.

Abbreviations: AD, Alzheimer's disease; *APOE*, apolipoprotein E; −, increased risk of outcome with higher protein levels; +, decreased risk of outcome with higher protein levels.

### 3.3 Cognitive function outcomes

**FIGURE 1:**
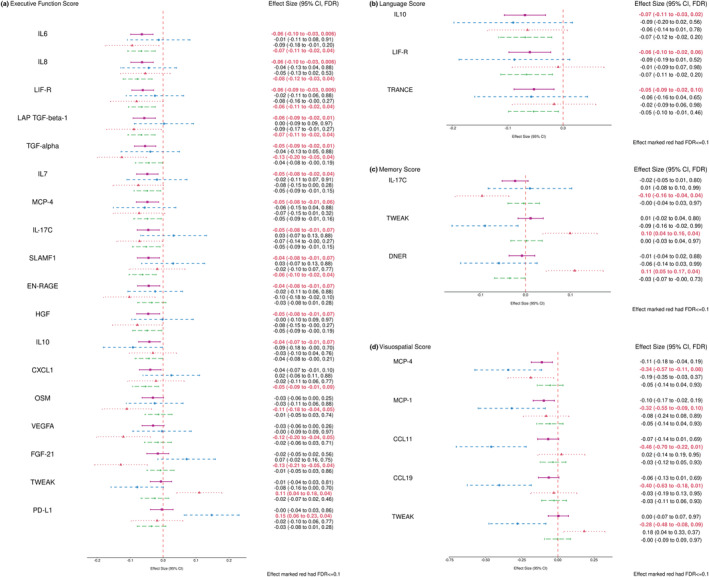
Forest plots of combined and stratified protein effect size for significant associations within four cognitive domains using Model 1 (FDR ≤ 0.1). (a) Proteins associated with executive function domain score; (b) Proteins associated with language domain score; (c) Proteins associated with memory domain score; (d) Proteins associated with visuospatial domain score. The covariates adjusted in Model 1 included: sex, age, education level, time in years between Exam 7 (blood sample) and cognitive testing date, a retest indicator, and *APOE* genotype. The pink square represented the combined sample (*N* = 708), the blue circle represented the ε2 Carriers (*N* = 87), the red triangle represented the ε4 Carriers (*N* = 133), and the green diamond represented the ε3ε3 subgroup (*N* = 468). *APOE*, apolipoprotein E; CI, confidence interval; FDR, false discovery rate.

**FIGURE 2:**
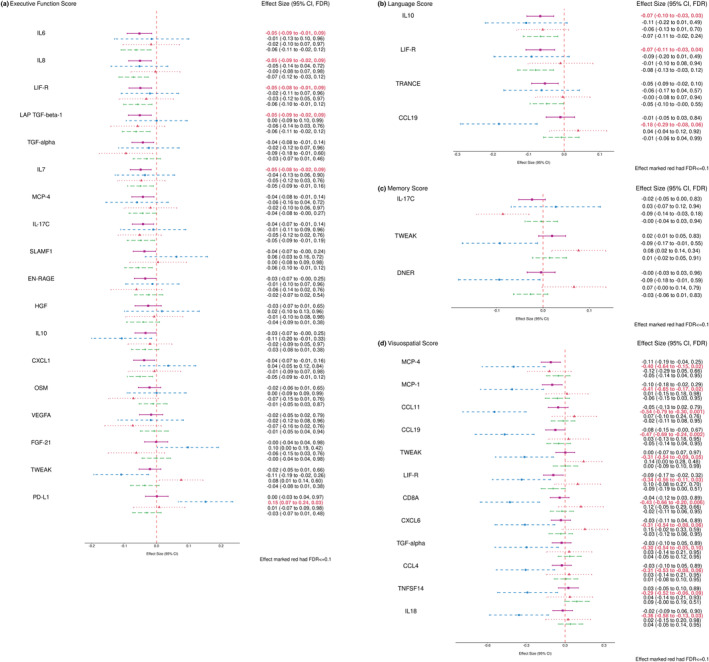
Forest plots of combined and stratified protein effect size for significant associations within four cognitive domains using Model 2 (FDR ≤ 0.1). (a) Proteins associated with executive function domain score; (b) Proteins associated with language domain score; (c) Proteins associated with memory domain score; (d) Proteins associated with visuospatial domain score. The covariates adjusted in Model 2 included: sex, age, education level, time in years between Exam 7 (blood sample) and cognitive testing date, a retest indicator, *APOE* genotype, indicators for prevalent cardiovascular diseases (CVD) such as prevalent stroke, prevalent CVD, and prevalent atrial fibrillation (AF) at Exam 7, and the CVD risk factors such as systolic and diastolic blood pressures (mmHg), diabetes status, treatment for hypertension, body‐mass index (kg/m^2^), current smoking status, total cholesterol level (mg/dL), high‐density lipoprotein cholesterol levels (HDL, measured in mg/dL), and use of lipid‐lowering agents at Exam 7. The pink square represented the combined sample (*N* = 708), the blue circle represented the ε2 Carriers (*N* = 87), the red triangle represented the ε4 Carriers (*N* = 133), and the green diamond represented the ε3ε3 subgroup (*N* = 468). APOE, apolipoprotein E, CI, confidence interval; FDR, false discovery rate.

Figure 1 provides a visual overview of the effects of proteins that have significant associations with one of the four domain scores in the full sample or one of the *APOE*‐genotype stratified subgroups. Figure 2 is a forest plot of the same associations and any additional new significant associations after adjustment for the Model 2 CVD and risk factor covariates. Results for analyses of all proteins with all domain scores and individual NP tests for all strata, using both Model 1 and Model 2 adjustments are provided in Table S1.

In the full sample, significant protein associations were observed with the EF and LAN domain factor scores. Higher levels of proteins IL6, IL8, LIF‐R, LAP TGF‐beta‐1, TGF‐alpha, IL7, MCP‐4, IL‐17C, SLAMF1, EN‐RAGE, HGF, and IL10 were significantly associated with lower EF factor scores, with effects ranging in size from −0.04 to −0.06 SD units per SD unit increment in protein level. Higher levels of IL10 and LIF‐R were also associated with lower LAN factor scores, along with TRANCE. There were no significant associations observed in the full sample in the MEM and VIS domains. The *APOE* ε3 stratum effect sizes were generally similar to those observed in the full sample, with CXCL1 and EF being the only association that met FDR significance in this group where they did not in the full sample. CXCL1, a pro‐inflammatory factor mediating neutrophil and monocyte infiltration (Wu et al., 2021), was no longer significant after adjusting for CVD risk factors.

The ε4 stratum effect size was in many cases more extreme than the effect sizes in the full sample for the EF domain. In the LAN domain, the ε4 stratum effect size was similar to the other strata for IL10, but for LIF‐R and TRANCE, ε4 associations were closer to 0 than for the other strata. Four proteins were associated with EF in the ε4 stratum but not in other strata: higher levels of OSM, VEGFA, and FGF‐21 were associated with lower EF scores, and higher levels of TWEAK were associated with higher EF scores. In the MEM domain, the only significant associations were observed in the ε4 stratum, where higher IL‐17C was associated with lower MEM scores, and higher TWEAK and DNER levels were associated with higher MEM scores.

The *APOE* ε2 stratum effect sizes were generally less extreme than the full sample effect size for the proteins associated with the EF domains in the full sample. Higher PD‐ L1 was associated with higher EF domain scores within the ε2 stratum, but not for other strata. The ε2 stratum had several significant associations in the VIS domain that were not significant for the other strata or the full sample: higher levels of MCP‐4, MCP‐1, CCL11, CCL19, and TWEAK were associated with lower VIS scores for ε2 carriers.

After adjusting for prevalent CVD and CVD risk factors (Model 2 covariates), the direction of effects for most proteins was the same, but most associations for the full sample, ε3 stratum, and ε4 stratum generally had attenuated effect sizes and lower association significance (Figure 2). LIF‐R remained significantly negatively associated with both the EF and LAN domain factor scores in the full sample. Notably, all significant associations observed in Model 1 for the ε2 stratum were more significant and all effect sizes were higher in magnitude when adjusting for the CVD and risk factor (Model 2) covariates, with the exception of the PD‐L1 association with the EF domain scores, for which significance and effect magnitude remained approximately the same. In the ε2 stratum, we observed seven additional significant associations for the VIS domain after adjusting for CVD and risk factor covariates: LIF‐R, CD8A, CXCL6, TGF‐alpha, CCL4, TNFSF14, and IL18, were all significantly associated with VIS domain scores in the ε2 stratum using Model 2 covariates, along with the original five proteins also associated in Model 1 (MCP‐4, MCP‐1, CCL11, CCL19, and TWEAK). All protein associations with VIS domain scores in the ε2 stratum were negative, that is, higher protein levels were associated with lower VIS scores.

#### 3.3.1 Sensitivity analyses

Sensitivity analyses were conducted when (1) excluding participants whose nearest NP exam was more than 2 years after Exam 7 (remaining *N* = 652), (2a) excluding 14 individuals with prevalent stroke, and (2b) excluding the participants with prevalent stroke, and additionally prevalent chronic leukemia or lymphoma, participants who reported use of glucocorticoids at Exam 7, and participants who were identified as outliers by PCA based on the 68 rank‐normalized proteins (remaining *N* = 654). The overall number of significant associations between cognitive function outcomes and proteins using Model 1 was lower, but effect direction and size were consistent with the primary analysis, indicating that the observed associations are robust to the time lapse between protein measurement and NP test and inclusion of potential outliers (Figures S2–S5). After excluding the 14 prevalent stroke participants we observed that the effect sizes were quite close to our current results and the standard deviations were slightly larger due to the reduction in sample size. For the EF domain in the ε4 stratum, Sensitivity Analysis 2b had somewhat larger effect sizes than we saw in the primary analysis, and more proteins were significantly associated, indicating that the outliers might attenuate some associations (Figure S2). Complete association results for the sensitivity analyses for all strata are provided in Tables S3 and S4.

### 3.4 Dementia outcomes

**FIGURE 3:**
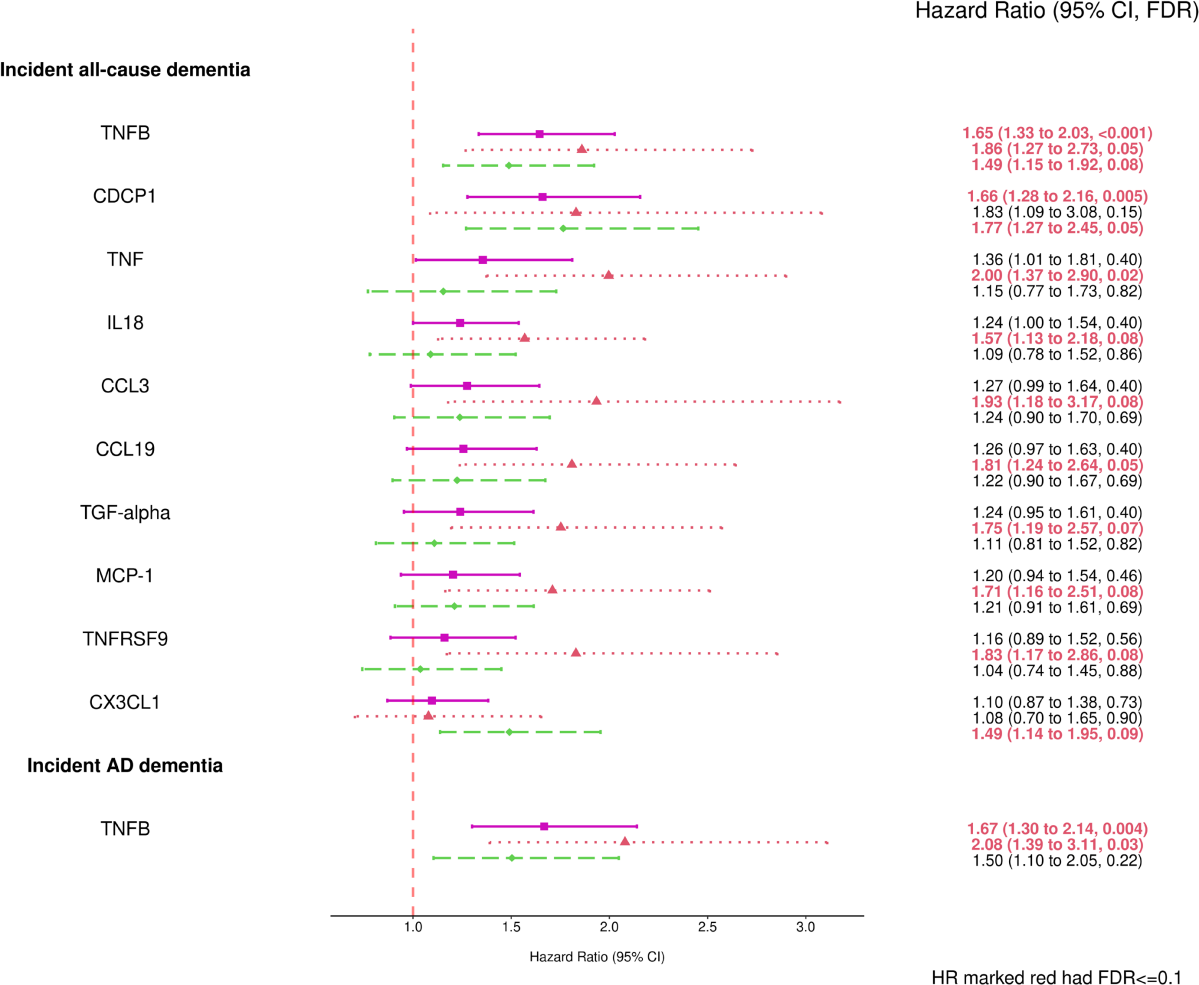
Forest plots of combined and stratified protein effect size for significant associations with dementia outcomes using Model 1 (FDR ≤ 0.1). The covariates adjusted in Model 1 included: sex, age, education level, and *APOE* genotype. The pink square represented the combined sample (*N* = 837), the red triangle represented the ε4 Carriers (*N* = 164), and the green diamond represented the ε3ε3 subgroup (*N* = 551). AD, Alzheimer's disease; APOE, apolipoprotein E; CI, confidence interval; FDR, false discovery rate; HR, hazard ratio.

**FIGURE 4:**
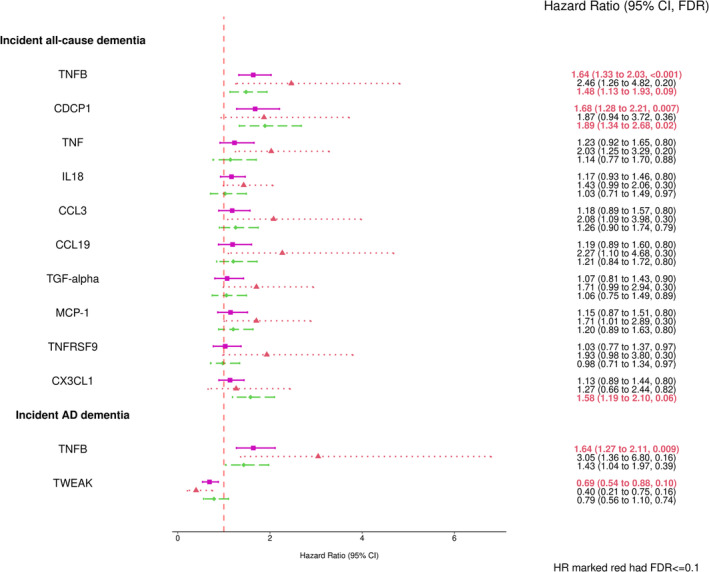
Forest plots of combined and stratified protein effect size for significant associations with dementia outcomes using Model 2 (FDR ≤ 0.1). The covariates adjusted in Model 2 included: sex, age, education level, *APOE* genotype, indicators for prevalent cardiovascular diseases (CVD) such as prevalent CVD, and prevalent atrial fibrillation (AF) at Exam 7, and the CVD risk factors such as systolic and diastolic blood pressures (mmHg), diabetes status, treatment for hypertension, body‐mass index (kg/m^2^), current smoking status, total cholesterol level (mg/dL), high‐density lipoprotein cholesterol levels (HDL, measured in mg/dL), and use of lipid‐lowering agents at Exam 7. The pink square represented the combined sample (*N* = 837), the red triangle represented the ε4 Carriers (*N* = 164), and the green diamond represented the ε3ε3 subgroup (*N* = 551). AD, Alzheimer's disease; APOE, apolipoprotein E; CI, confidence interval; FDR, false discovery rate; HR, hazard ratio.

The significant associations identified by the primary analyses in the full sample adjusting for Model 1 covariates (sex, age, education level, and *APOE* genotype) and using the full follow‐up time are shown in Figure 3. The median follow‐up time for incident all‐cause and AD dementia were 18.2 and 18.3 years, and there were 87 and 64 events, respectively. The sample characteristics for the age‐restricted sample are shown in Table S2b. Higher levels of TNFB and CDCP1 were associated with higher risks of incident dementia, with a hazard ratio (HR) of 1.65 per unit increment in TNFB level (95% CI = (1.33, 2.03), FDR < 0.001) and HR of 1.66 per unit increment in CDCP1 level (95% CI = (1.28, 2.16), FDR = 0.005). TNFB was also significantly associated with higher risks of incident AD dementia (HR = 1.67, 95% CI = (1.30, 2.14), FDR = 0.004). TNFB and CDCP1 were not associated with any of the cognitive domain scores in the full sample or any subgroup (all FDR > 0.1). After adjusting for Model 2 CVD and risk factor covariates, the associations were still observed with similar HRs but slightly lower significance (see Figure 4). Higher levels of TNFB and CDCP1 remained significantly associated with higher risks of incident dementia, with HR of 1.64 per unit increase in TNFB level (95% CI = (1.33, 2.03), FDR <0.001) and HR of 1.68 per unit increase in CDCP1 level (95% CI = (1.28, 2.21), FDR = 0.007). TNFB was still associated with incident AD dementia (HR = 1.64, 95% CI = (1.28, 2.11), FDR = 0.009). In contrast, another circulating factor, TWEAK, became significantly associated with a lower risk of incident AD dementia in the full sample after adjusting for the CVD and risk factor covariates (HR = 0.69, 95% CI = (0.54, 0.88), FDR = 0.10).

There were 10 or fewer events of incident all‐cause dementia or AD dementia in ε2 carriers within the dementia sample (see Table S2b), and thus the stratified analyses included only the ε4 stratum and ε3 stratum (Figure 3). In the ε3 stratum, TNFB, CDCP1, and CX3CL1 were significantly associated with incident dementia. In the ε4 stratum, eight proteins including the protein TNFB were associated with higher risks of dementia (HR > 1), and TNFB was also associated with higher risks of AD dementia (HR > 1, FDR = 0.03). Of the eight proteins associated with dementia, TGF‐alpha was also associated with lower EF domain scores in the ε4 stratum (FDR = 0.04) and full sample (FDR = 0.01), and CCL19 was also associated with lower VIS domain scores in the ε2 stratum (FDR = 0.01); all other proteins were not associated with any cognitive domain score in ε4 or any other stratum (all FDR > 0.1).

In the secondary analyses adjusting for Model 2 covariates (see Figure 4) the ε4 stratum associations with incident dementia were smaller and were not statistically significant, while we did observe significant associations in the full sample and the ε3 stratum. The association of TNFB with incident AD dementia for ε4 stratum was also attenuated after accounting for Model 2 covariates. Results for analyses of all proteins with incident all‐cause and AD dementia for all strata, using both Model 1 and Model 2 adjustments are provided in Tables 2 and 3.

#### 3.4.1 Sensitivity analyses

After excluding participants younger than age 60 at Exam 7 (*N* = 444, 85 and 64 events for incident all‐cause and AD dementia, respectively), the effect sizes for TNFB and CDCP1 associations remained similar. TWEAK was significantly associated with a lower risk of incident all‐cause and AD dementia in the combined sample. TNF remained associated with higher risks of incident dementia in the ε4 stratum and CXCL1 was associated with higher risks of incident dementia in the ε3 stratum (see Figure S6). Complete association results for this sensitivity analysis of all strata are provided in Table S5.

## 4 DISCUSSION

We observed several significant associations in cross‐sectional analyses between 68 proteins included in the OLINK inflammatory panel and cognitive domain scores and in prospective analyses of incident all‐cause and AD dementia in the community‐based FHS Offspring cohort. First, more of the inflammatory proteins were associated with the EF domain than any other cognitive domain, and looking across all strata of analyses, several proteins were associated with more than one cognitive domain, including IL10 (EF and LAN), LIF‐R (EF, LAN, VIS), TWEAK (EF, MEM, VIS), CCL19 (LAN and VIS), IL‐17C (EF, MEM), and MCP‐4 and TGF‐alpha (EF and VIS). Second, stratified analyses identified some potential differences in inflammatory protein effects, particularly between the *APOE* ε2 and ε4 carriers. *APOE* ε4 has several biologic roles leading to the development of dementia including affecting inflammation and atherosclerosis (Bonomini et al., 2010), while *APOE* ε2 is the protective variant for dementia with the protective biologic mechanisms remaining to be elucidated (Kim et al., 2022). ε2 carriers have reduced risks of CVD and hypertension and lower lipid levels (Kuo et al., 2020). The associations observed for the ε4 carrier subgroup were with memory and executive function domain scores, and appeared to be smaller when adjusting for CVD and risk factors. For the ε2 carrier group, most of the significant associations were for visuospatial domain scores, and these associations were stronger with adjustment for CVD and the risk factors. Third, we observed that most of the inflammatory proteins associated with cognitive scores were not associated with incident all‐cause dementia or AD dementia, and similarly the proteins associated with the dementia outcomes were not associated with the cross‐sectional cognitive scores, suggesting that cross‐sectional measures of cognition at the time of blood draw reflect lifelong or mid to late life cognitive processes that are different from cognitive impairment and dementia from neurodegeneration. However, there were some inflammatory protein associations with concordant observations. For example, among ε4 carriers, higher levels of TWEAK, also known as tumor necrosis factor (ligand) superfamily member 12, were associated with better memory domain scores and higher levels of TWEAK were associated with a lower risk for incident AD dementia in the full sample in the model adjusting for CVD and risk factors. It is notable that the effect of TWEAK on cognition and dementia among ε4 carriers was protective, unlike the majority of protein associations observed in this study.

Deficits in executive function have been observed in persons with vascular cognitive impairments (Gorelick et al., 2011). Vascular brain injury leads to a spectrum of cognitive impairments from mild cognitive deficits to vascular dementia, the second most common cause of dementia after AD (Dichgans & Leys, 2017). Further, small vessel disease of the brain contributes to more than fifty percent of dementia worldwide, including in cases that also have AD pathology (Sweeney et al., 2019). Hence, addressing vascular risk factors is important in optimizing brain health (Gorelick et al., 2017) and lowering risks for cognitive decline and dementia (Debette et al., 2011; Llewellyn et al., 2008; Pase et al., 2016). Circulating inflammatory biomarkers are associated with higher cardiovascular risk, and there is increasing interest in therapeutics used to target residual inflammation with the goal to decrease adverse outcomes (Aday & Ridker, 2019). In this study, we observed 12 inflammatory biomarker associations with EF domain scores in the full sample (IL‐6, IL‐7, IL‐8, IL‐10, IL‐17C, LIF‐R, LAP TGF beta‐1, TGF‐alpha, MCP‐4, SLAMF1, ENRAGE, and HGF), five biomarker associations in ε4 carriers and one biomarker association in ε2 carriers. The associations were attenuated but many remained statistically significant in the full sample (IL‐6, IL‐7, IL‐8, LIF‐R, and LAP TGF‐beta‐1) after adjustment for cardiovascular disease and risk factors, suggesting additional biologic pathways play a role in these associations. Others have reported associations between IL‐6 and cognitive function independent of risk factors in a racially/ethnically diverse sample (Economos et al., 2013) and in older community‐dwelling women. Unlike IL‐6, IL‐10 is an anti‐inflammatory cytokine. Consistent with our observations, higher levels of IL‐10 were significantly associated with poorer performances in executive function in older adults from the Berlin Aging Study II (Tegeler et al., 2016). Using an IL‐10 expressing APP mouse model, investigators observed an unexpected negative effect of IL‐10 on cognition and Aβ proteostasis (Chakrabarty et al., 2015). Further investigation is needed to determine if blocking IL‐10 may have beneficial effects on cognitive outcomes. Experimental work has shown that LIF‐R activation has neuroprotective functions including enhancing neural cell survival and reducing inflammatory responses to brain injury (Davis & Pennypacker, 2018). Our observation that higher levels of soluble LIF‐R in the peripheral circulation were associated with lower executive function and language domain scores are consistent with the hypothesis that the soluble form of the receptor potentially acts as a non‐signaling decoy. HGF (hepatocyte growth factor) is a neurotrophic factor with effects on angiogenesis and has been associated with small vessel disease in persons with cognitive impairment and AD (Zhu et al., 2018). Consistent with our findings, HGF was associated with EF in the Northern Manhattan Study (Elkind et al., 2021).

*APOE* is linked to longevity (Deelen et al., 2019), cardiovascular disease, and neurodegenerative disorders including AD and related dementias (Ashford & Mortimer, 2002; Blacker et al., 1997; Kunkle et al., 2019). The biologic functions of *APOE* that may relate to the development of dementia include its role in lipid metabolism and atherosclerosis, maintaining the integrity of the blood–brain barrier and blood–nerve barrier, and modulating inflammation with interactions with macrophages and T cells as well as microglia and astrocytes (Zhang et al., 2011). Cytokines are critical to the neuroinflammatory process and *APOE* effects on neuroinflammation may be through its interactions with cytokines. Evidence suggest cytokine levels may differ by *APOE* genotype with some pro‐inflammatory cytokines higher in ε4 carriers and others lower (Duarte‐Guterman et al., 2020). Among ε4 carriers only we observed associations with memory domain scores (IL‐17C, TWEAK, DNER) and we observed some associations with executive function domain scores only in ε4 carriers (TWEAK, FGF‐21, OSM, VEGFA). The ε4 carrier associations with the executive function and memory domains were attenuated after adjustment for CVD and CVD risk factors, suggesting that the underlying mechanism may involve vascular pathways. OSM is a pleiotropic cytokine and a member of the IL‐6 family of cytokines involved in a broad array of biological processes including inflammation and vascular dysfunction (Stawski & Trojanowska, 2019), and may contribute to neuroinflammation through dysfunction of the blood–brain barrier (Hermans et al., 2022). OSM has also been reported to induce angiogenesis by increasing VEGF secretion (Vasse et al., 1999). VEGFA (vascular endothelial growth factor A) is involved in angiogenesis and neurogenesis with neuroprotective effects in *APOE* ε4 mice (Salomon‐Zimri et al., 2016). In contrast, but consistent with our findings of higher VEGFA levels associated with lower executive function scores, higher VEGFA gene expression was associated with worse global cognition in ε4 carriers from the Religious Orders Study, but the association was not present when accounting for multiple hypothesis testing (Moore et al., 2020). The association of VEFGA with cognitive outcomes is complex. In the Esther study, VEGFA was associated with vascular dementia with associations stronger in ε4 negative participants (Trares et al., 2022). We observed that higher levels of TWEAK were associated with both higher executive function and memory domain scores in ε4 carriers, but not other *APOE* genotype groups. TWEAK is also involved in vascular processes including angiogenesis and proliferation of endothelial cells. In animal models of neuropsychiatric lupus, TWEAK contributed to the disruption of the blood–brain barrier and neuronal damage with hippocampal gliosis (Wen et al., 2015).

The *APOE* ε2 allele is associated with a lower risk for AD with individuals homozygous for ε2 at especially low risk (Reiman et al., 2020). Reports of associations of ε2 with cognitive function however have largely been inconsistent due to different study designs and small samples of ε2 carriers (Kim et al., 2022). At least one study reported ε2 carriers had better verbal memory and fluency, but only in women. Interestingly, sex‐specific effects of ε2 were observed for lipids and the inflammatory marker C‐reactive protein (Lamonja‐Vicente et al., 2021). A second study conducted in centenarians identified proteins associated with the preservation of cognitive function associated with ε2 allele (Sebastiani et al., 2019). In contrast to the ε4 associations, we observed that among ε2 carriers only, higher levels of CCL11 (also known as eotaxin), CCL19, MCP‐1, MCP‐4, and TWEAK were associated with lower visuospatial scores and the associations appeared to be independent of CVD and CVD risk factors, with additional protein associations observed with adjustment for CVD and CVD risk factors. CCL11, CCL19, MCP‐1, and MCP‐4 are pro‐inflammatory chemokines that have been implicated in neuroinflammatory processes (Bettcher et al., 2019; Larsson et al., 2015; Le Page et al., 2015; Villeda et al., 2011). In animal models CCL11 plasma levels correlate with lower hippocampal neurogenesis and CCL11 levels have been reported to increase in both plasma and cerebral spinal fluid of healthy older adults (Villeda et al., 2011). CCL11 in young mice also impaired memory and learning, implicating blood borne factors in aging‐related illness (Villeda et al., 2011). In the Northern Manhattan Study, CCL11 was associated with several cognitive domains and overall cognitive function (Elkind et al., 2021) and in the FRAILOMIC consortium, CCL11 was negatively associated with cognitive performance in rural dwelling adults (Butcher et al., 2018). The *APOE* genotype was not investigated in these prior studies. We also observed among ε2 carriers that higher levels of PD‐L1, an immune cell checkpoint ligand, were associated with higher executive function domain scores. Immune checkpoint blockade with anti‐PD‐1 and anti‐PD‐ligand antibodies in cancer immunotherapy have been successful and well tolerated. It has been proposed that the immune tolerance and immune system checkpoints needed to fight tumor growth leaves cancer survivors with lower risk for AD (Rogers et al., 2020). In mouse models of AD, PD‐1 blockade treatment reduced Aβ plaque and improved memory (Baruch et al., 2016). More work is needed to determine if immune checkpoint blockade could be a potential therapeutic for AD and related dementias and whether *APOE* carrier status modifies benefit (Schwartz et al., 2019). Our data in ε2 carriers with high levels of soluble, circulating PD‐L1 associating with higher EF scores may indicate that soluble PD‐L1 acts as a decoy receptor blocking the PD‐1/PD‐L1 interaction, much like anti‐PD‐L1 antibodies do in cancer immunotherapy.

This study identified two inflammatory biomarkers associated with incident dementia (TNFB and CDCP1) and one (TNFB) was also associated with incident AD dementia in the full sample and among ε4 carriers. In addition, TWEAK was associated with AD dementia in the model that adjusted for CVD and its risk factors and many additional inflammatory markers were associated with dementia in ε4 carriers, but the association was not significant with adjustment for CVD and cardiovascular risk factors. TNFB is a pleiotropic cytokine also known as lymphotoxin‐alpha and is produced by T cells and leukocytes and secreted by a number of cells including astrocytes and endothelial cells. One small study of Chinese patients with AD and healthy controls noted an association of TNFB with cognitive function in patients with AD (Lu et al., 2022). Our findings differ from several other studies of proteomic or inflammatory panels in part due to different study designs and assay panels used (Soares et al., 2012; Trares et al., 2022; Whelan et al., 2019). The Biomarkers Consortium in AD Plasma Proteomics Project identified different biomarkers than our study, but consistent with our study noted distinct biomarker profiles in ε4 carriers (Soares et al., 2012). Importantly, that study established that plasma biomarkers could improve the specificity in differentiating AD versus healthy controls. The ESTHER study used the same OLINK inflammatory panel used in our study and reported that 80.6% of biomarkers tested were associated with incident dementia and about 30% of biomarkers were associated with AD (Trares et al., 2022). Four biomarker clusters were identified (CX3CL1, ENRAGE, LAP TGF‐beta‐1, VEGFA) and all associations were stronger among those with no ε4 alleles. Our study profiled the OLINK inflammatory panel in a single batch and did not observe the same high correlations between biomarkers.

In another study based on 11 inflammatory protein biomarkers measured in the Systems Approach to Biomarker Research in Cardiovascular Disease, we found that higher levels of CD40L and myeloperoxidase (MPO) were associated with poor performance in NP tests regarding executive function (Fang, Doyle, Chen, et al., 2022). It only assessed *APOE* ε4 carrier status and individual NP tests rather than the cognitive factor scores in the three *APOE* strata. Hence, we have expanded the scope of association studies between inflammatory biomarkers and cognitive outcomes in this study.

Our study has several strengths. We leveraged the community‐based FHS Offspring cohort that was deeply phenotyped for cognitive aging with neuropsychological testing at regular intervals and was well characterized for CVD risk factors. The calibrated cognitive factor scores summarizing different cognitive tests for three domains on the same scale increased the power of associations. Our sample was dementia‐free at baseline and has since been followed over 20 years in a prospective manner, with dementia review administered by a standardized protocol. Stored plasma samples at Exam 7 allowed for comprehensive profiling using the OLINK inflammation panels. Finally, the *APOE* genotype status allowed us to examine associations by *APOE* ε2 and ε4 carrier status the most common genetic risk factors associated with risk or protection from AD (Reiman et al., 2020).

Our study has several limitations that merit comment. First, the modest sample size of our study limited the power to detect significant associations, particularly within *APOE* ε2 carriers for incident all‐cause and AD dementia outcomes. Second, our analyses were cross‐sectional, and cannot be used to infer causality. Third, the cognitive domain scores reflect shared variance across all test items assigned to a cognitive domain, but may not sufficiently capture specific cognitive performance within a domain (e.g., verbal episodic retrieval within the memory domain). Fourth, the blood draw used for biomarkers profiling and the exam dates for the NP tests were not contemporaneous, and the distance between these differed across participants. While we accounted for this time‐lapse in our analyses as a covariate, this time difference may have decreased the strength of some associations between the proteins and cognitive domain scores, biasing our findings toward the null. Fifth, inflammatory cytokines may degrade with time even at ‐80C, and time differences in storage across participants may create differences in protein levels that are hard to detect. Sixth, our study measured inflammatory protein levels in plasma at a single time point. While these blood samples are easy to obtain, they may not reflect the levels in the brain. Seventh, the FHS Offspring cohort participants are primarily white, well‐educated, and reside in New England. Replication in independent samples that are ethnically and geographically diverse is required to confirm our findings.

In conclusion, several circulating inflammatory proteins have been shown to associate with cognitive domain scores. Stratified analyses suggested differences in protein effects between *APOE* ε2 and ε4 carriers, with most ε4 carrier associations with the executive function and memory domains, and most ε2 associations with the visuospatial domain. Higher levels of TNFB and CDCP1 were associated with an increased risk of incident all‐cause and AD dementia.

## AUTHOR CONTRIBUTIONS

JC performed data curation, formal analysis, investigation, and visualization, wrote the original draft and wrote for revisions and editing. MFD, KLL, and JMM performed conceptualization, funding acquisition, data curation, investigation, and visualization, wrote the original draft, and wrote revisions and editing. PKC and PS performed analyses of the cognitive data to produce domain scores, and wrote revisions and editing. YF, JM, CLS, MLA, and WQQ performed the investigation and wrote for review and editing.

## FUNDING INFORMATION

This work was supported by the National Institutes of Health R01AG067457. Support for collection of FHS data was provided by the National Heart, Lung, and Blood Institute and the Boston University School of Medicine, Framingham Heart Study (contract number 75N92019D00031). The dementia outcomes and neuropsychological testing data were collected with funding from the National Institute Aging and of Neurological Disorders and Stroke (R01‐AG054076, R01‐AG016495, R01‐AG008122, R01‐AG033040, R01‐NS017950).

## CONFLICT OF INTEREST STATEMENT

The authors declare that they have no conflict of interest.

## Supporting information


Appendix S1.

Appendix S2.

## Data Availability

Framingham Heart Study Offspring data are available for request via NHLBI Biological Specimen and Data Repositories Information Coordinating Center (BioLINCC) https://biolincc.nhlbi.nih.gov/studies/framoffspring/.
