# Supplementary material for: Peripheral inflammatory biomarkers are associated with cognitive function and dementia: Framingham Heart Study Offspring cohort
Source: Aging Cell. 2023 Aug 16;22(10):e13955. doi: 10.1111/acel.13955 (PMC10577533; doi:10.1111/acel.13955)
Supplement: Supplementary file 1 — Appendix S1. [file ACEL-22-e13955-s001.docx]

Contents

**Files:**

Supplementary File: Full set of association results for all 68 proteins with the 4 domain scores and individual cognitive test scores, and for the two incident outcomes

Tab 1: Model 1 NP results, Model 2 NP results side by side

Tab 2: Model 1 dementia outcomes results, Model 2 dementia outcomes results side by side

Tab 3: Model 1 NP results for sensitivity analysis 1

Tab 4: Model 1 NP results for sensitivity analysis 2

Tab 5: Model 1 dementia outcomes results for sensitivity analysis 3

**Figures:**

Figure S1: Study sample inclusion and exclusion description

Figure S2-S5: Bar plots of changes in effect from primary analysis for the sensitivity analyses for four domains

Figure S6: Forest plots of combined and stratified protein effect size for significant associations with dementia outcomes using Model 1 on the subsamples with age at Exam 7 older than 60 years old

**Tables:**

Table S1: Full list of proteins showing the corresponding LOD and proportion of observations below LOD

Table S2:

1. The mean and SD of the 68 proteins in the NP/factor score, and dementia samples
2. Summary of cognitive domain scores, neuropsychological (NP) test scores and dementia outcomes

Table S3: Summary of significant associations for cognitive function outcomes using Model 2

Table S4: Summary of significant associations for dementia outcomes using Model 2

879 FHS Offspring participants who attended exam 7 between 1998 and 2001 and had an available plasma sample for biomarker profiling

10 Were excuded:

2 due to missing biomarker levels and 8 due to missing *APOE* genotype

869 Participants with protein panel and complete *APOE* status

161 Were excluded due to no neuropsychological tests within 5 years after Exam 7

32 Were excluded:

17 with prevalent stroke and

15 with missing covariate or outcome data

837 Participants in incident all-cause and AD dementia sample

708 Participants with cross-sectional neuropsychological testing outcomes and cognitive factor scores

Figure S1: Study sample inclusion and exclusion description

Note: FHS = Framingham Heart Study, APOE = Apolipoprotein E, AD = Alzheimer’s Disease.

Table S1: Full list of proteins showing the corresponding LOD and proportion with missing values or values below LOD

| Uniprot ID | Protein | Protein full name | LOD | Missing proportion | Below LOD Proportion |
| --- | --- | --- | --- | --- | --- |
| Q13541 | 4E-BP1 | Eukaryotic translation initiation factor 4E-binding protein 1 | 1.857 | 0.001 | 0.000 |
| P00813 | ADA | Adenosine deaminase | 1.472 | 0.002 | 0.000 |
| Q5T4W7 | ARTN | Artemin | 1.677 | 0.001 | 0.955 |
| O15169 | AXIN1 | Axin-1 | 1.421 | 0.002 | 0.024 |
| P01138 | Beta-NGF | Beta-nerve growth factor | -0.231 | 0.001 | 0.989 |
| Q14790 | CASP-8 | Caspase-8 | 1.577 | 0.001 | 0.033 |
| P51671 | CCL11 | Eotaxin | 2.118 | 0.001 | 0.000 |
| Q99731 | CCL19 | C-C motif chemokine 19 | 2.100 | 0.001 | 0.000 |
| P78556 | CCL20 | C-C motif chemokine 20 | 2.531 | 0.001 | 0.000 |
| P55773 | CCL23 | C-C motif chemokine 23 | 2.391 | 0.002 | 0.000 |
| O15444 | CCL25 | C-C motif chemokine 25 | 2.324 | 0.001 | 0.000 |
| Q9NRJ3 | CCL28 | C-C motif chemokine 28 | 1.284 | 0.001 | 0.000 |
| P10147 | CCL3 | C-C motif chemokine 3 | 1.650 | 0.001 | 0.000 |
| P13236 | CCL4 | C-C motif chemokine 4 | 1.904 | 0.001 | 0.000 |
| Q9BZW8 | CD244 | Natural killer cell receptor 2B4 | 2.450 | 0.001 | 0.000 |
| P25942 | CD40 | Tumor necrosis factor receptor superfamily member 5 | 3.540 | 0.001 | 0.000 |
| P06127 | CD5 | T-cell surface glycoprotein CD5 | 1.575 | 0.001 | 0.000 |
| P30203 | CD6 | T-cell differentiation antigen CD6 | 2.178 | 0.001 | 0.000 |
| P01732 | CD8A | T-cell surface glycoprotein CD8 alpha chain | 2.679 | 0.002 | 0.000 |
| Q9H5V8 | CDCP1 | CUB domain-containing protein 1 | 0.335 | 0.001 | 0.000 |
| P09603 | CSF-1 | Macrophage colony-stimulating factor 1 | 2.270 | 0.001 | 0.000 |
| P28325 | CST5 | Cystatin-D | 0.337 | 0.001 | 0.000 |
| P78423 | CX3CL1 | Fractalkine | 1.946 | 0.001 | 0.001 |
| P09341 | CXCL1 | Growth-regulated alpha protein | 2.674 | 0.001 | 0.000 |
| P02778 | CXCL10 | C-X-C motif chemokine 10 | 2.806 | 0.001 | 0.000 |
| O14625 | CXCL11 | C-X-C motif chemokine 11 | 1.569 | 0.001 | 0.000 |
| P42830 | CXCL5 | C-X-C motif chemokine 5 | 2.138 | 0.001 | 0.000 |
| P80162 | CXCL6 | C-X-C motif chemokine 6 | 2.031 | 0.001 | 0.001 |
| Q07325 | CXCL9 | C-X-C motif chemokine 9 | 1.965 | 0.002 | 0.000 |
| Q8NFT8 | DNER | Delta and Notch-like epidermal growth factor-related receptor | 0.969 | 0.001 | 0.000 |
| P80511 | EN-RAGE | Protein S100-A12 | 1.881 | 0.001 | 0.002 |
| O95750 | FGF-19 | Fibroblast growth factor 19 | 1.825 | 0.001 | 0.000 |
| Q9NSA1 | FGF-21 | Fibroblast growth factor 21 | 2.436 | 0.002 | 0.002 |
| Q9GZV9 | FGF-23 | Fibroblast growth factor 23 | 2.174 | 0.001 | 0.941 |
| P12034 | FGF-5 | Fibroblast growth factor 5 | 1.617 | 0.001 | 0.725 |
| P49771 | Flt3L | Fms-related tyrosine kinase 3 ligand | 2.962 | 0.001 | 0.000 |
| P39905 | GDNF | Glial cell line-derived neurotrophic factor | 2.377 | 0.001 | 0.912 |
| P14210 | HGF | Hepatocyte growth factor | 2.144 | 0.001 | 0.000 |
| P01579 | IFN-gamma | Interferon gamma | 3.610 | 0.001 | 0.001 |
| P01583 | IL-1 alpha | Interleukin-1 alpha | -0.437 | 0.001 | 0.970 |
| Q13651 | IL-10RA | Interleukin-10 receptor subunit alpha | 1.512 | 0.001 | 0.694 |
| Q08334 | IL-10RB | Interleukin-10 receptor subunit beta | 2.616 | 0.001 | 0.000 |
| P29460 | IL-12B | Interleukin-12 subunit beta | 1.433 | 0.001 | 0.000 |
| Q13261 | IL-15RA | Interleukin-15 receptor subunit alpha | 1.814 | 0.001 | 0.347 |
| Q16552 | IL-17A | Interleukin-17A | 2.839 | 0.004 | 0.532 |
| Q9P0M4 | IL-17C | Interleukin-17C | 2.316 | 0.001 | 0.053 |
| Q13478 | IL-18R1 | Interleukin-18 receptor 1 | 2.390 | 0.001 | 0.000 |
| Q9NYY1 | IL-20 | Interleukin-20 | 1.557 | 0.002 | 0.968 |
| Q9UHF4 | IL-20RA | Interleukin-20 receptor subunit alpha | 1.913 | 0.001 | 0.907 |
| Q8N6P7 | IL-22 RA1 | Interleukin-22 receptor subunit alpha-1 | 3.381 | 0.001 | 0.976 |
| Q13007 | IL-24 | Interleukin-24 | 3.052 | 0.013 | 0.924 |
| P14784 | IL-2RB | Interleukin-2 receptor subunit beta | 1.802 | 0.001 | 0.938 |
| P22301 | IL10 | Interleukin-10 | 1.741 | 0.001 | 0.001 |
| P35225 | IL13 | Interleukin-13 | 1.729 | 0.002 | 0.938 |
| Q14116 | IL18 | Interleukin-18 | 2.346 | 0.001 | 0.000 |
| P60568 | IL2 | Interleukin-2 | 2.413 | 0.001 | 0.995 |
| O95760 | IL33 | Interleukin-33 | 2.372 | 0.001 | 0.987 |
| P05112 | IL4 | Interleukin-4 | 1.754 | 0.001 | 0.861 |
| P05113 | IL5 | Interleukin-5 | 2.406 | 0.001 | 0.866 |
| P05231 | IL6 | Interleukin-6 | 2.580 | 0.001 | 0.139 |
| P13232 | IL7 | Interleukin-7 | 1.371 | 0.002 | 0.010 |
| P10145 | IL8 | Interleukin-8 | 1.023 | 0.001 | 0.000 |
| P01137 | LAP TGF-beta-1 | Transforming growth factor beta-1 proprotein | 1.434 | 0.001 | 0.000 |
| P15018 | LIF | Leukemia inhibitory factor | 1.048 | 0.001 | 0.980 |
| P42702 | LIF-R | Leukemia inhibitory factor receptor | 1.737 | 0.001 | 0.000 |
| P13500 | MCP-1 | C-C motif chemokine 2 | 3.542 | 0.001 | 0.000 |
| P80075 | MCP-2 | C-C motif chemokine 8 | 2.838 | 0.001 | 0.000 |
| P80098 | MCP-3 | C-C motif chemokine 7 | 1.945 | 0.003 | 0.695 |
| Q99616 | MCP-4 | C-C motif chemokine 13 | 4.943 | 0.001 | 0.000 |
| P03956 | MMP-1 | Interstitial collagenase | 3.402 | 0.001 | 0.001 |
| P09238 | MMP-10 | Stromelysin-2 | 2.072 | 0.001 | 0.000 |
| Q99748 | NRTN | Neurturin | 1.658 | 0.001 | 0.943 |
| P20783 | NT-3 | Neurotrophin-3 | 2.263 | 0.002 | 0.110 |
| O00300 | OPG | Tumor necrosis factor receptor superfamily member 11B | 2.405 | 0.001 | 0.000 |
| P13725 | OSM | Oncostatin-M | 0.874 | 0.001 | 0.000 |
| Q9NZQ7 | PD-L1 | Programmed cell death 1 ligand 1 | 2.723 | 0.001 | 0.000 |
| P21583 | SCF | Kit ligand | 2.442 | 0.001 | 0.000 |
| Q8IXJ6 | SIRT2 | NAD-dependent protein deacetylase sirtuin-2 | 3.273 | 0.001 | 0.885 |
| Q13291 | SLAMF1 | Signaling lymphocytic activation molecule | 2.410 | 0.002 | 0.231 |
| P50225 | ST1A1 | Sulfotransferase 1A1 | 1.114 | 0.001 | 0.543 |
| O95630 | STAMBP | STAM-binding protein | 1.238 | 0.001 | 0.000 |
| P01135 | TGF-alpha | Protransforming growth factor alpha | 0.493 | 0.001 | 0.002 |
| P01375 | TNF | Tumor necrosis factor | 1.040 | 0.001 | 0.000 |
| P01374 | TNFB | Lymphotoxin-alpha | 1.428 | 0.001 | 0.000 |
| Q07011 | TNFRSF9 | Tumor necrosis factor receptor superfamily member 9 | 2.048 | 0.001 | 0.000 |
| O43557 | TNFSF14 | Tumor necrosis factor ligand superfamily member 14 | 2.301 | 0.001 | 0.001 |
| P50591 | TRAIL | Tumor necrosis factor ligand superfamily member 10 | 1.687 | 0.001 | 0.000 |
| O14788 | TRANCE | Tumor necrosis factor ligand superfamily member 11 | 1.565 | 0.001 | 0.000 |
| Q969D9 | TSLP | Thymic stromal lymphopoietin | 1.654 | 0.001 | 0.888 |
| O43508 | TWEAK | Tumor necrosis factor ligand superfamily member 12 | 1.196 | 0.001 | 0.000 |
| P00749 | uPA | Urokinase-type plasminogen activator | 2.632 | 0.001 | 0.000 |
| P15692 | VEGFA | Vascular endothelial growth factor A | 3.300 | 0.003 | 0.000 |

Note: LOD = limit of detection.

Table S2 (a): The mean and SD of the 68 proteins in the NP/factor score, and dementia samples

| Protein | NP/Factor Score Sample n=708 | | Dementia Sample  n=837 | |
| --- | --- | --- | --- | --- |
|  | **Mean** | **SD** | **Mean** | **SD** |
| 4E-BP1 | 6.50 | 0.69 | 6.50 | 0.70 |
| ADA | 5.26 | 0.34 | 5.26 | 0.33 |
| AXIN1 | 2.97 | 0.75 | 2.98 | 0.74 |
| CASP-8 | 2.14 | 0.32 | 2.14 | 0.31 |
| CCL11 | 7.55 | 0.47 | 7.55 | 0.48 |
| CCL19 | 8.72 | 0.91 | 8.71 | 0.91 |
| CCL20 | 6.99 | 1.01 | 6.98 | 1.01 |
| CCL23 | 10.19 | 0.47 | 10.20 | 0.47 |
| CCL25 | 6.48 | 0.58 | 6.49 | 0.59 |
| CCL28 | 2.38 | 0.36 | 2.38 | 0.36 |
| CCL3 | 5.91 | 0.55 | 5.92 | 0.55 |
| CCL4 | 5.91 | 0.60 | 5.93 | 0.62 |
| CD244 | 5.99 | 0.32 | 5.99 | 0.32 |
| CD40 | 10.77 | 0.33 | 10.77 | 0.34 |
| CD5 | 6.06 | 0.38 | 6.07 | 0.38 |
| CD6 | 5.74 | 0.47 | 5.74 | 0.46 |
| CD8A | 9.27 | 0.67 | 9.29 | 0.67 |
| CDCP1 | 2.84 | 0.58 | 2.86 | 0.59 |
| CSF-1 | 9.50 | 0.23 | 9.50 | 0.23 |
| CST5 | 5.63 | 0.48 | 5.63 | 0.48 |
| CX3CL1 | 3.86 | 0.39 | 3.86 | 0.39 |
| CXCL1 | 7.87 | 1.02 | 7.86 | 1.01 |
| CXCL10 | 9.17 | 0.87 | 9.15 | 0.86 |
| CXCL11 | 6.85 | 0.74 | 6.86 | 0.76 |
| CXCL5 | 8.46 | 1.41 | 8.45 | 1.42 |
| CXCL6 | 7.57 | 0.78 | 7.57 | 0.76 |
| CXCL9 | 6.94 | 0.83 | 6.95 | 0.84 |
| DNER | 8.09 | 0.26 | 8.09 | 0.26 |
| EN-RAGE | 2.75 | 0.53 | 2.76 | 0.53 |
| FGF-19 | 7.93 | 0.92 | 7.95 | 0.93 |
| FGF-21 | 5.74 | 1.16 | 5.75 | 1.17 |
| Flt3L | 8.83 | 0.44 | 8.83 | 0.43 |
| HGF | 8.71 | 0.45 | 8.72 | 0.46 |
| IFN-gamma | 6.29 | 0.93 | 6.30 | 0.94 |
| IL-10RB | 6.91 | 0.29 | 6.91 | 0.29 |
| IL-12B | 7.20 | 0.62 | 7.19 | 0.63 |
| IL-15RA | 1.93 | 0.24 | 1.93 | 0.25 |
| IL-17C | 3.22 | 0.68 | 3.23 | 0.69 |
| IL-18R1 | 7.76 | 0.42 | 7.76 | 0.42 |
| IL10 | 3.43 | 0.64 | 3.43 | 0.62 |
| IL18 | 8.86 | 0.56 | 8.87 | 0.56 |
| IL6 | 3.36 | 0.81 | 3.36 | 0.80 |
| IL7 | 2.16 | 0.52 | 2.15 | 0.52 |
| IL8 | 3.88 | 0.61 | 3.89 | 0.61 |
| LAP TGF-beta-1 | 6.64 | 0.34 | 6.64 | 0.34 |
| LIF-R | 3.46 | 0.23 | 3.46 | 0.23 |
| MCP-1 | 11.49 | 0.40 | 11.50 | 0.42 |
| MCP-2 | 8.83 | 0.58 | 8.83 | 0.59 |
| MCP-4 | 12.56 | 0.64 | 12.56 | 0.63 |
| MMP-1 | 11.85 | 1.05 | 11.82 | 1.04 |
| MMP-10 | 8.91 | 0.66 | 8.91 | 0.65 |
| NT-3 | 2.59 | 0.29 | 2.60 | 0.30 |
| OPG | 10.06 | 0.39 | 10.07 | 0.39 |
| OSM | 2.83 | 0.85 | 2.81 | 0.84 |
| PD-L1 | 5.36 | 0.35 | 5.36 | 0.35 |
| SCF | 9.05 | 0.45 | 9.04 | 0.47 |
| SLAMF1 | 2.68 | 0.38 | 2.68 | 0.39 |
| STAMBP | 3.49 | 0.47 | 3.49 | 0.47 |
| TGF-alpha | 2.26 | 0.41 | 2.26 | 0.39 |
| TNF | 3.86 | 0.50 | 3.86 | 0.49 |
| TNFB | 4.55 | 0.47 | 4.56 | 0.47 |
| TNFRSF9 | 5.81 | 0.52 | 5.81 | 0.52 |
| TNFSF14 | 3.68 | 0.45 | 3.67 | 0.45 |
| TRAIL | 7.42 | 0.29 | 7.41 | 0.29 |
| TRANCE | 4.47 | 0.59 | 4.46 | 0.58 |
| TWEAK | 8.20 | 0.30 | 8.20 | 0.31 |
| uPA | 9.93 | 0.33 | 9.93 | 0.33 |
| VEGFA | 10.31 | 0.29 | 10.31 | 0.29 |

Note: NP = Neuropsychological, SD = standard deviation.

Table S2 (b): Summary of cognitive domain scores, inverse-normal transformed NP test scores and dementia outcomes

| **NP/Factor Score Sample**  **n=708** | | | | | | | | | | | | | |
| --- | --- | --- | --- | --- | --- | --- | --- | --- | --- | --- | --- | --- | --- |
|  | | **ε2 carriers**  **n=87** | | | **ε4 carriers**  **n=133** | | | **ε3ε3**  **n=468** | | | **All**  **n=708** | | |
|  | | **Mean** | **SD** | | **Mean** | | **SD** | **Mean** | | **SD** | **Mean** | | **SD** |
| **Age at exam 7** | | 59.9 | 9.5 | | 61.5 | | 8.6 | 61.2 | | 9.3 | 61.1 | | 9.1 |
| **Retest proportion** | | 0 | | | 0.015 | | | 0.021 | | | 0.020 | | |
| **Domain Scores** | |  |  | |  | |  |  | |  |  | |  |
| **EF domain factor score** | | 0.319 | 0.498 | | 0.368 | | 0.548 | 0.382 | | 0.512 | 0.368 | | 0.519 |
| **LAN domain factor score** | | 0.512 | 0.515 | | 0.549 | | 0.540 | 0.548 | | 0.554 | 0.543 | | 0.548 |
| **MEM domain factor score** | | 0.574 | 0.437 | | 0.559 | | 0.457 | 0.571 | | 0.430 | 0.563 | | 0.435 |
| **VIS score (HVOT)** | | -0.016 | 1.110 | | 0.047 | | 0.985 | -0.017 | | 0.979 | -0.002 | | 0.991 |
| **Individual NP test scores** | |  | | | | | | | | | | | |
| **SIM** | | -0.048 | 0.897 | | 0.036 | | 1.010 | -0.001 | | 1.010 | -0.001 | | 0.992 |
| **TRAILSBA** | | 0.074 | 1.130 | | 0.028 | | 1.010 | -0.040 | | 0.969 | 0.000 | | 1.000 |
| **BNT30** | | -0.122 | 0.923 | | 0.032 | | 0.904 | -0.023 | | 0.934 | -0.026 | | 0.930 |
| **LMD** | | 0.161 | 1.040 | | -0.019 | | 1.030 | -0.009 | | 0.965 | 0.000 | | 0.991 |
| **VRD** | | -0.140 | 0.988 | | 0.025 | | 0.996 | 0.014 | | 0.961 | -0.005 | | 0.977 |
| **PASD** | | -0.004 | 0.965 | | -0.011 | | 0.913 | -0.032 | | 0.901 | -0.026 | | 0.906 |
| **Dementia Sample (Full)**  **n=837** | | | | | | | | | | | | | |
| **Dementia-related Outcomes** | **Median follow-up Time** | | | **ε2 carriers**  **n=102** | | **ε4 carriers**  **n=164** | | | **ε3ε3**  **n=551** | | | **All**  **n=837** | |
| **Incident all-cause dementia** | 18.18 | | | 7 | | 27 | | | 50 | | | 87 | |
| **Incident AD dementia** | 18.27 | | | 4 | | 20 | | | 37 | | | 64 | |
| **Dementia Sample (>60 years old)**  **n= 444** | | | | | | | | | | | | | |
| **Dementia-related Outcomes** | **Median**  **follow-up Time** | | | **ε2 carriers**  **n=46** | | **ε4 carriers**  **n=90** | | | **ε3ε3**  **n=296** | | | **All**  **n=444** | |
| **Incident all-cause dementia** | 16.26 | | | 7 | | 27 | | | 48 | | | 85 | |
| **Incident AD dementia** | 17.06 | | | 4 | | 21 | | | 36 | | | 64 | |

Note: NP = Neuropsychological, SD = standard deviation, EF = executive function, LAN = language, MEM = memory, VIS = visuospatial, HVOT = Hooper Visual Organization Test, SIM = Similarities subtest, TRAILSBA = Trails Making test B - Trails Making test A, BNT30 = 30 - item Boston Naming Test, LMD = Logical Memory - delayed recall, VRD = Visual Reproduction - delayed recall, PASD = Paired Association learning - delayed recall, AD = Alzheimer’s Disease.

Table S3: Summary of significant associations for cognitive function outcomes using Model 2 in full sample and by *APOE* status strata

| Domain | Full sample | ε2 stratum | ε4 stratum | ε3 stratum |
| --- | --- | --- | --- | --- |
| **Executive Function** | $-$: 5 | $+$: 1 |  |  |
| Language | $-:$ 2 | $-:$ 1 |  |  |
| Memory |  |  |  |  |
| Visuospatial |  | $-:$ 12 |  |  |

Note: Model 2 covariates included: sex, age, education level, time in years between Exam 7 (blood sample) and cognitive testing date, a retest indicator, *APOE* genotype, indicators for prevalent cardiovascular diseases (CVD) such as prevalent stroke, prevalent CVD, and prevalent atrial fibrillation (AF) at Exam 7, and the CVD risk factors such as systolic and diastolic blood pressures (mmHg), diabetes status, treatment for hypertension, body-mass index (kg/m2), current smoking status, total cholesterol level (mg/dL), high-density lipoprotein cholesterol levels (HDL, measured in mg/dL), and use of lipid-lowering agents at Exam 7.

APOE = Apolipoprotein E.

Table S4: Summary of significant associations for dementia outcomes using Model 2 in full sample and by *APOE* status strata

| Outcome | Full sample | ε4 stratum | ε3 stratum |
| --- | --- | --- | --- |
| **Incident all-cause dementia** | $-$: 2 |  | $-$: 3 |
| **Incident AD dementia** | $+$: 1  $-$: 1 |  |  |

Note: Model 2 covariates included: sex, age, education level, *APOE* genotype, indicators for prevalent cardiovascular diseases (CVD) such as prevalent CVD, and prevalent atrial fibrillation (AF) at Exam 7, and the CVD risk factors such as systolic and diastolic blood pressures (mmHg), diabetes status, treatment for hypertension, body-mass index (kg/m2), current smoking status, total cholesterol level (mg/dL), high-density lipoprotein cholesterol levels (HDL, measured in mg/dL), and use of lipid-lowering agents at Exam 7.

APOE = Apolipoprotein E, AD = Alzheimer’s Disease.

-: increased risk of outcome with higher protein levels; +: decreased risk of outcome with higher protein levels.

Figure S2: Bar plots of changes in effect from primary analysis for the sensitivity analyses for Executive Function domain

(a)
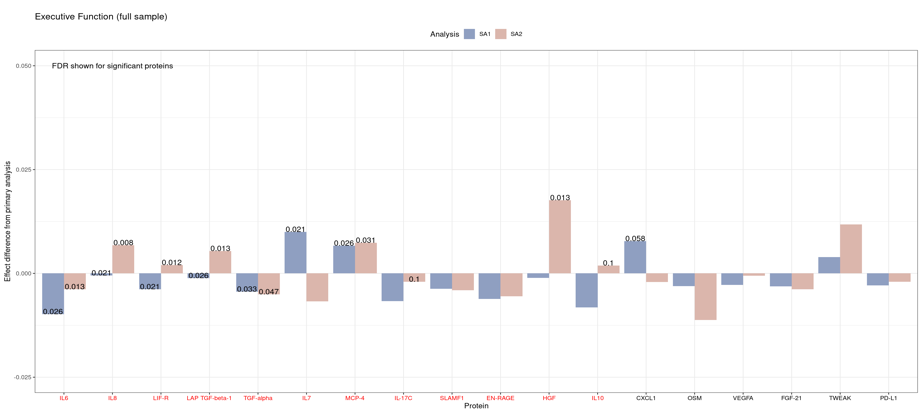


(b)
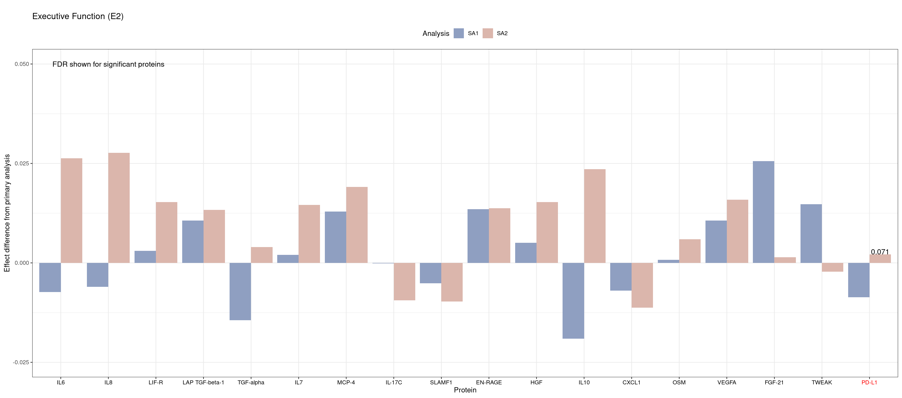


(c)
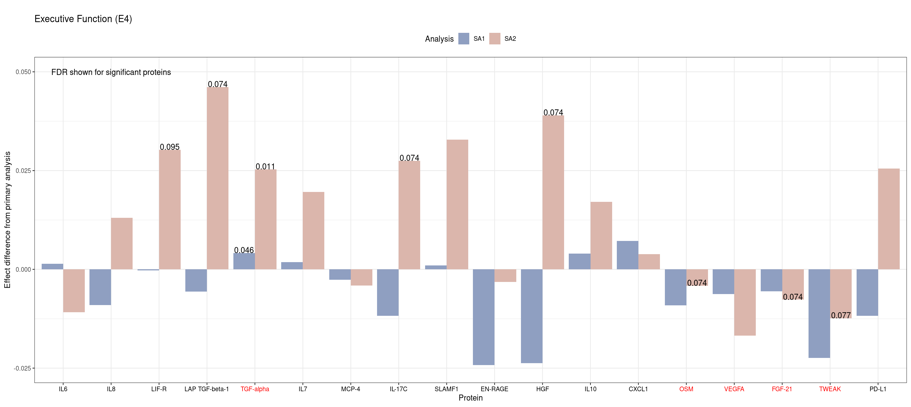


(d)
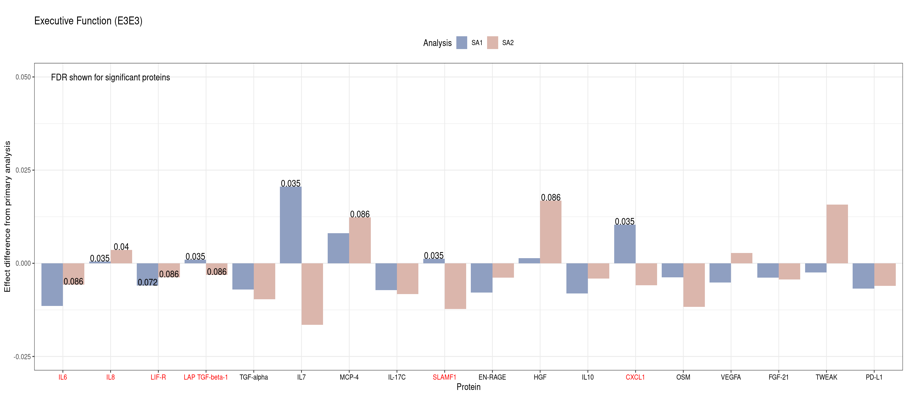


Note: The x-axis showed all significant proteins (FDR≤0.1 either in the full sample or three *APOE* strata) in the primary analysis and those marked red were significant in the primary analysis. Y-axis showed the change in the absolute effect of each sensitivity analysis from the primary analysis. Proteins significant in the sensitivity analysis had the FDR marked. In SA1, the associations between cognitive function outcomes and proteins were evaluated through Model 1 including only the participants who underwent NP tests within two years after Exam 7 (N=652). SA2 assessed whether some specific participants drove the NP associations and investigated analyses through Model 1 excluding participants with prevalent stroke, prevalent chronic leukemia or lymphoma, who reported use of glucocorticoids at Exam 7, and who were identified as outliers by principal component analysis (PCA) based on the 68 rank-normalized proteins (N=654). Model 1 covariates included: sex, age, education level, time in years between Exam 7 (blood sample) and cognitive testing date, a retest indicator, and *APOE* genotype. (a) presented the results in the full sample, (b)(c)(d) presented stratified analyses results in three *APOE* strata.

SA = sensitivity analysis, FDR = false discovery rate, APOE = Apolipoprotein E.

Figure S3: Bar plots of changes in effect from primary analysis for the sensitivity analyses for Language domain

(a)
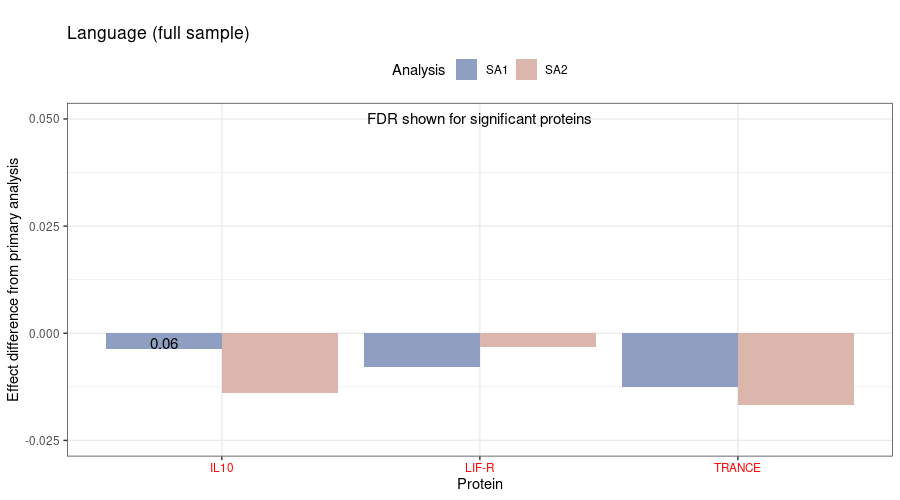


(b)
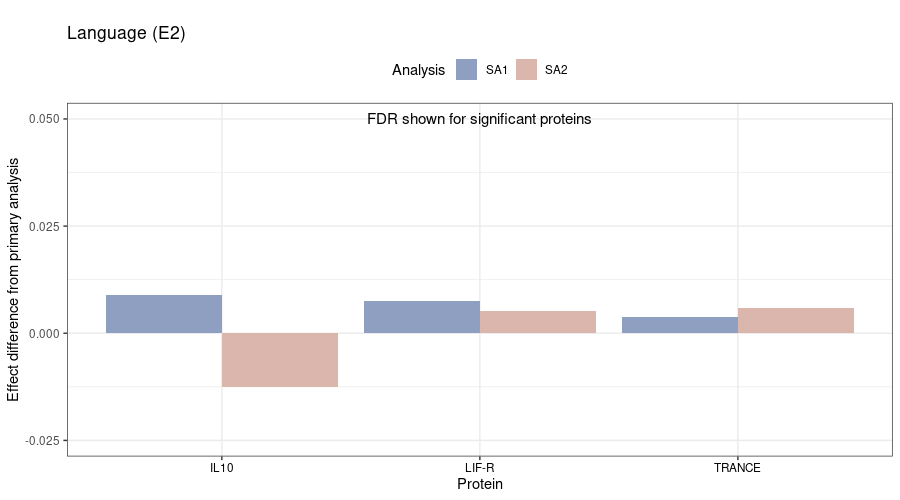


(c)
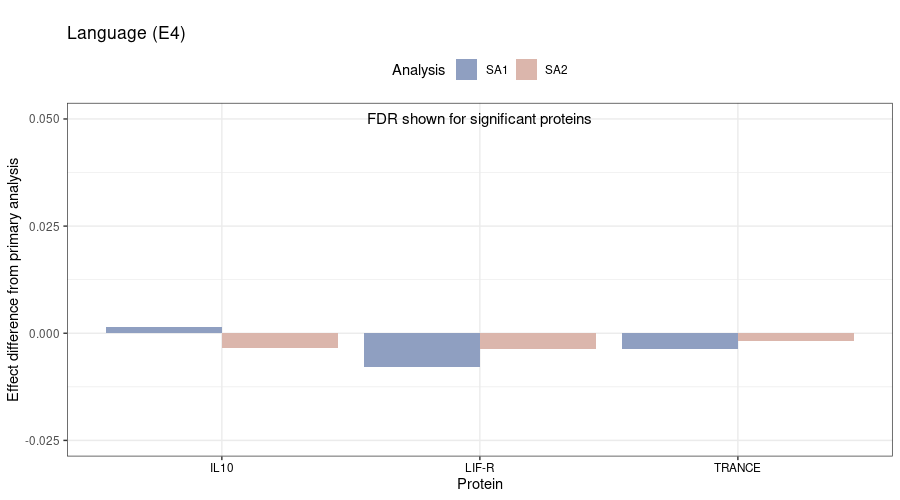


(d)
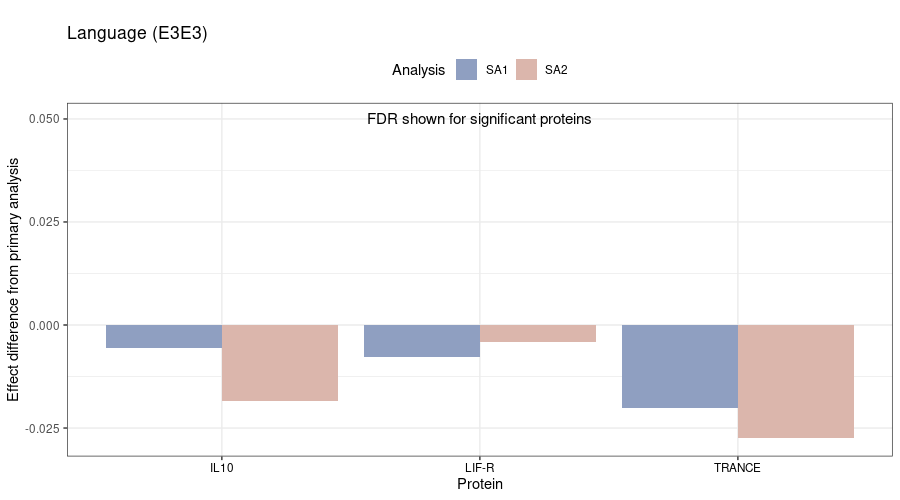


Note: The x-axis showed all significant proteins (FDR≤0.1 either in the full sample or three *APOE* strata) in the primary analysis and those marked red were significant in the primary analysis. Y-axis showed the change in the absolute effect of each sensitivity analysis from the primary analysis. Proteins significant in the sensitivity analysis had the FDR marked. In SA1, the associations between cognitive function outcomes and proteins were evaluated through Model 1 including only the participants who underwent NP tests within two years after Exam 7 (N=652). SA2 assessed whether some specific participants drove the NP associations and investigated analyses through Model 1 excluding participants with prevalent stroke, prevalent chronic leukemia or lymphoma, who reported use of glucocorticoids at Exam 7, and who were identified as outliers by principal component analysis (PCA) based on the 68 rank-normalized proteins (N=654). Model 1 covariates included: sex, age, education level, time in years between Exam 7 (blood sample) and cognitive testing date, a retest indicator, and *APOE* genotype. (a) presented the results in the full sample, (b)(c)(d) presented stratified analyses results in three *APOE* strata.

SA = sensitivity analysis, FDR = false discovery rate, APOE = Apolipoprotein E.

Figure S4: Bar plots of changes in effect from primary analysis for the sensitivity analyses for Memory domain

1.
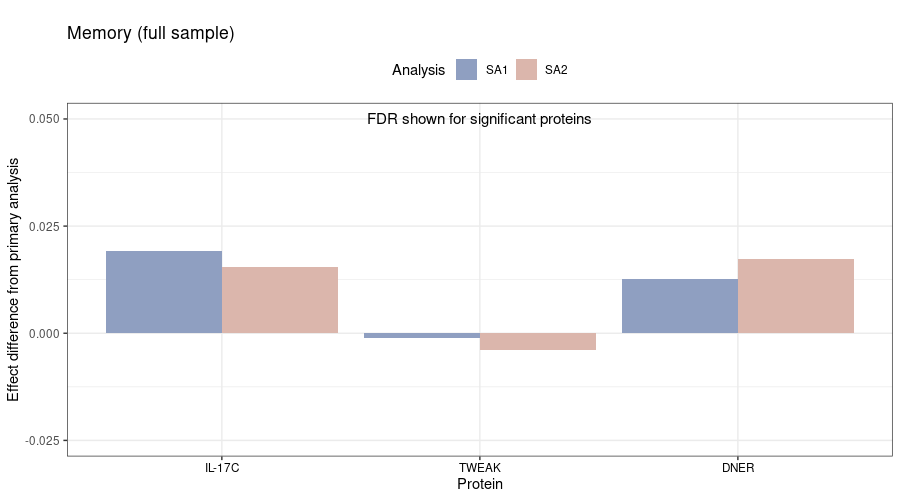

2.
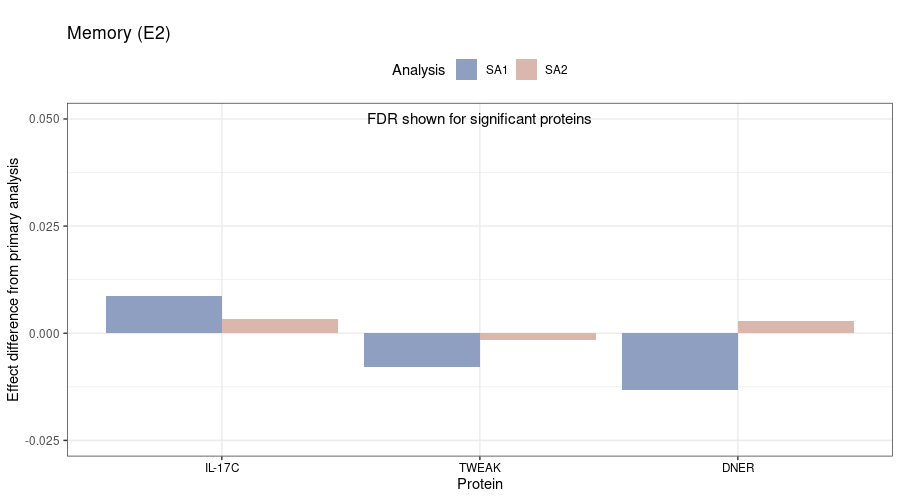


(c)
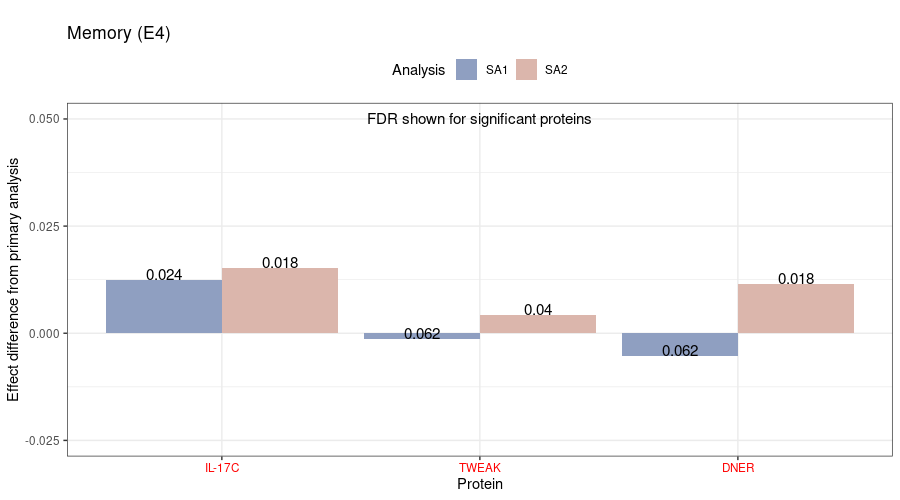


(d)
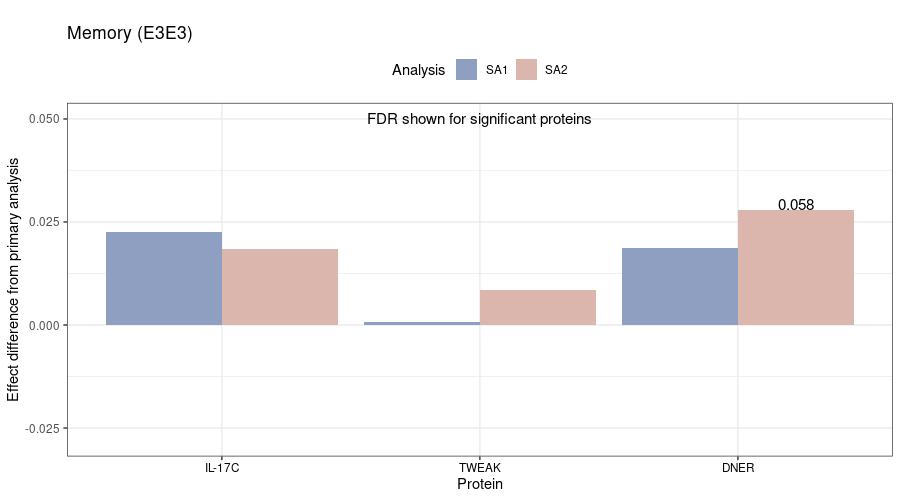


Note: The x-axis showed all significant proteins (FDR≤0.1 either in the full sample or three *APOE* strata) in the primary analysis and those marked red were significant in the primary analysis. Y-axis showed the change in the absolute effect of each sensitivity analysis from the primary analysis. Proteins significant in the sensitivity analysis had the FDR marked. In SA1, the associations between cognitive function outcomes and proteins were evaluated through Model 1 including only the participants who underwent NP tests within two years after Exam 7 (N=652). SA2 assessed whether some specific participants drove the NP associations and investigated analyses through Model 1 excluding participants with prevalent stroke, prevalent chronic leukemia or lymphoma, who reported use of glucocorticoids at Exam 7, and who were identified as outliers by principal component analysis (PCA) based on the 68 rank-normalized proteins (N=654). Model 1 covariates included: sex, age, education level, time in years between Exam 7 (blood sample) and cognitive testing date, a retest indicator, and *APOE* genotype. (a) presented the results in the full sample, (b)(c)(d) presented stratified analyses results in three *APOE* strata.

SA = sensitivity analysis, FDR = false discovery rate, APOE = Apolipoprotein E.

Figure S5: Bar plots of changes in effect from primary analysis for the sensitivity analyses for Visuospatial domain

1.
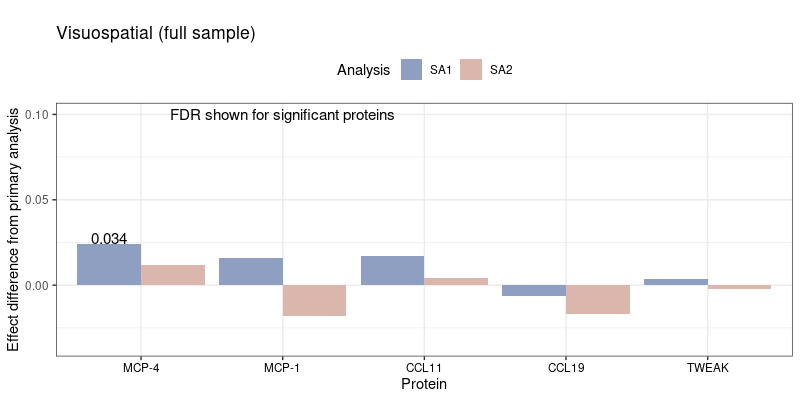

2.
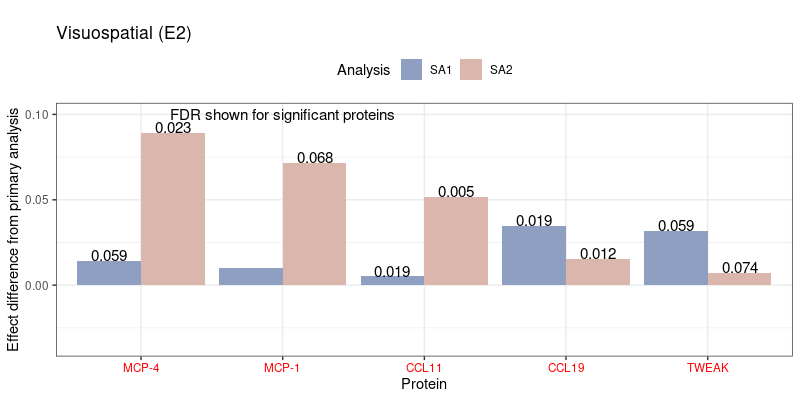

3.
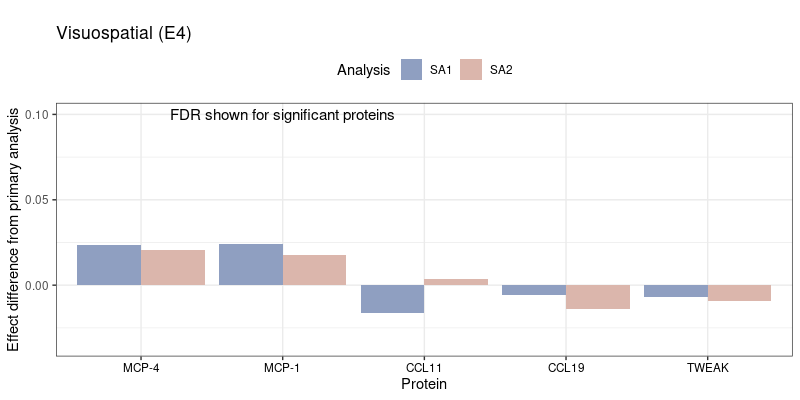

4.
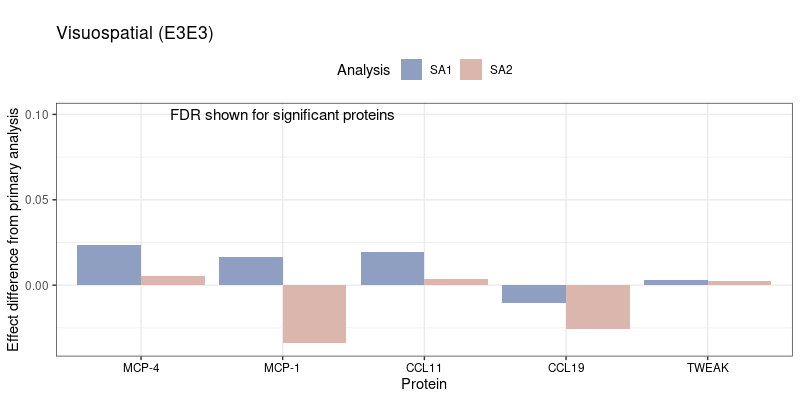


Note: The x-axis showed all significant proteins (FDR≤0.1 either in the full sample or three *APOE* strata) in the primary analysis and those marked red were significant in the primary analysis. Y-axis showed the change in the absolute effect of each sensitivity analysis from the primary analysis. Proteins significant in the sensitivity analysis had the FDR marked. In SA1, the associations between cognitive function outcomes and proteins were evaluated through Model 1 including only the participants who underwent NP tests within two years after Exam 7 (N=652). SA2 assessed whether some specific participants drove the NP associations and investigated analyses through Model 1 excluding participants with prevalent stroke, prevalent chronic leukemia or lymphoma, who reported use of glucocorticoids at Exam 7, and who were identified as outliers by principal component analysis (PCA) based on the 68 rank-normalized proteins (N=654). Model 1 covariates included: sex, age, education level, time in years between Exam 7 (blood sample) and cognitive testing date, a retest indicator, and *APOE* genotype. (a) presented the results in the full sample, (b)(c)(d) presented stratified analyses results in three *APOE* strata.

SA = sensitivity analysis, FDR = false discovery rate, APOE = Apolipoprotein E.

Figure S6: Forest plots of combined and stratified protein effect size for significant associations with dementia outcomes using Model 1 on the subsamples with age at Exam 7 older than 60 years old (FDR ≤0.1)


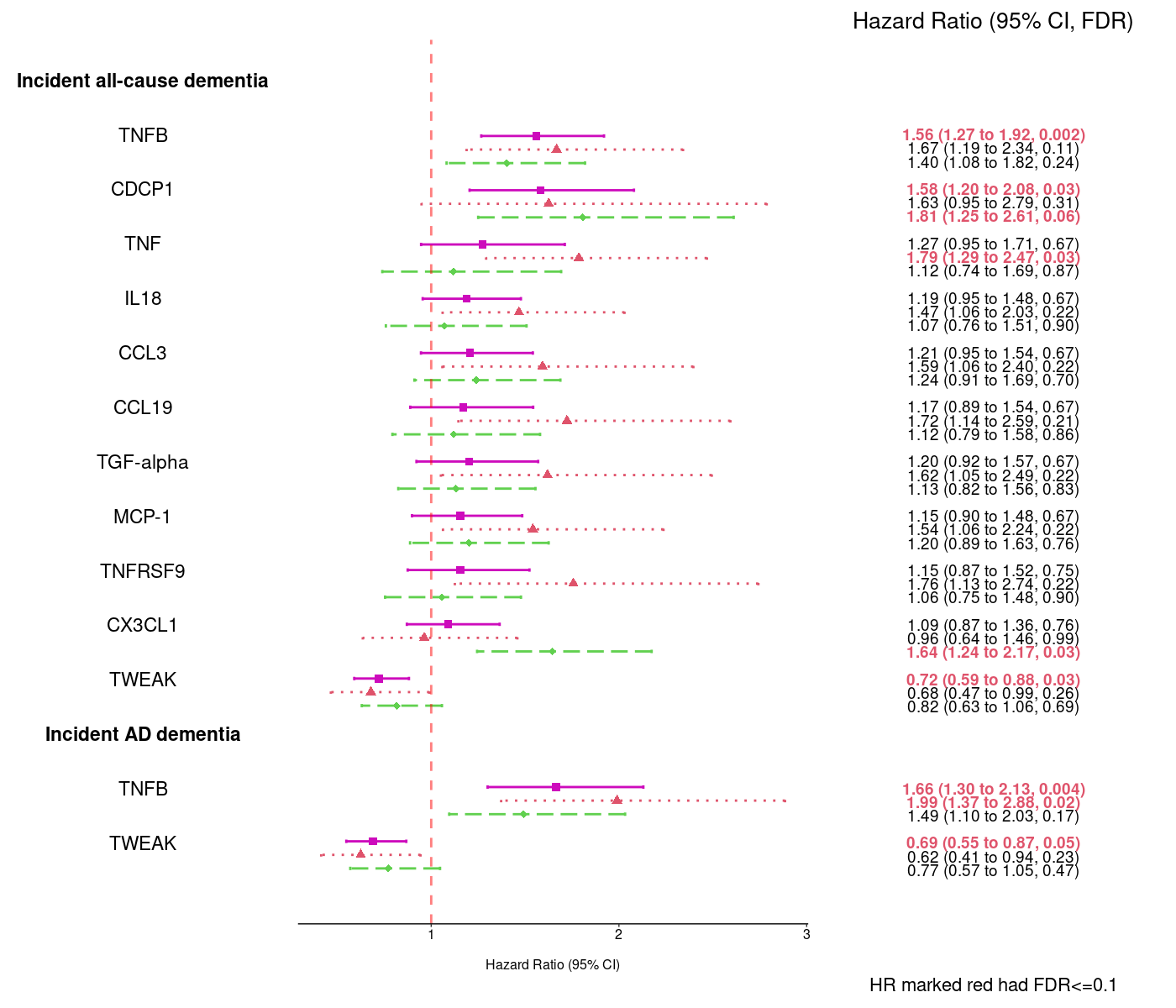


Note: The covariates adjusted in Model 1 included: sex, age, education level, and *APOE* genotype. The pink square represented the combined sample (N=444), the red triangle represented the ε4 Carriers (N=90), and the green diamond represented the ε3ε3 subgroup (N=296).

FDR= false discovery rate, CI = confidence interval, AD = Alzheimer’s Disease, APOE = Apolipoprotein E, HR = hazard ratio.
